# Long-term exposure to polyethylene restructures the multi-kingdom soil microbiota in maize fields

**DOI:** 10.1038/s42003-025-08899-8

**Published:** 2025-11-24

**Authors:** Zhen Shi, Li Xiong, Zhaojie Li, Farooq Shah, Xin Zhou, Qianhua Yuan, Bao-Luo Ma, Wei Wu

**Affiliations:** 1 School of Breeding and Multiplication (Sanya Institute of Breeding and Multiplication), Hainan University, Sanya, China; 2 School of Tropical Agriculture and Forestry, Hainan University, Haikou, China; 3 Department of Agronomy, Abdul Wali Khan University Mardan, Khyber, Pakhtunkhwa Pakistan; 4 State Key Laboratory of Microbial Diversity and Innovative Utilization, Institute of Microbiology, Chinese Academy of Sciences, Beijing, China; 5 Ottawa Research and Development Centre, Agriculture and Agri-Food Canada, Ottawa, Canada

**Keywords:** Microbial ecology, Microbial ecology

## Abstract

Soil contamination from polyethylene (PE) has emerged as a new global concern, yet its long–term legacy effects on soil microbiota remain poorly understood. Here, we conduct an eight–year field experiment to investigate how PE residues influence microbiota assembly across multiple microbial kingdoms (bacteria, fungi, and protists), and the consequent effects on soil antibiotic resistome. Our results reveal that bacterial communities are more stable and resilient than fungal and protistan communities in response to PE exposure. Bacterial assembly is predominantly shaped by deterministic processes under PE exposure, unlike the more stochastic patterns observed in the other domains. This bacterial deterministic assembly coincides with enhanced microbial biodegradation potential, evidenced by increased abundance of carbon–cycling functional genes in the plastisphere. In parallel, antibiotic resistance genes (ARGs) are found to be more prevalent in the plastisphere under PE exposure. While bacterial hosts play a dominant role in ARGs dissemination, fungal and protistan taxa also contribute through broader inter–kingdom ecological interactions. Together, these findings highlight the critical importance of considering multi–kingdom microbiota assembly when assessing the environmental risks of plastic pollution.

## Abstract (web summary)

Bacterial hosts are identified as the main drivers of antibiotic resistance gene (ARG) dissemination under long-term polyethylene exposure, with fungi and protists contributing through cross-kingdom interactions.

## Introduction

Plastic films have become an integral component of current cropping systems owing to their key role in improving soil microclimate and crop productivity^1,2^. Among them, Polyethylene (PE) is one of the most common and widely used plastic films in farmland due to its attractive properties such as affordability, flexibility, and easy processing^3–5^. However, its poor biodegradability and incomplete recovery after use have led to widespread soil contamination, exacerbating the global threat of plastic pollution^6,7^. Currently, it is estimated that nearly 90% of plastic waste (6.3 billion metric tons) is accumulated in landfills or the natural environment, with only 10% being recycled^8^. Over time, large amounts of plastic film fragments are incorporated into the soil, where macroplastics gradually decompose into microplastics (MPs), posing long-term risks to soil health^9–11^.

The increasing load of PE residues has rendered terrestrial ecosystems as vulnerable to plastic pollution as aquatic environments, hence leading to heightened global attention^12^. As a result, a growing number of studies have begun to examine how PE residues affect soil microbial communities, particularly bacteria and fungi^13–15^. In our previous field experiment with a shorter PE duration than the present study, we demonstrated that PE residues can create a unique microbial habitat known as the plastisphere, which serves as a selective niche, altering bacterial community structure and function. However, that study primarily focused on bacterial community and short-term PE exposure, while overlooking the complex interactions among different microbial groups, particularly the role of protists^16^. Protists, as microbial eukaryotes, act as key predators and ecosystem regulators, playing a pivotal role in shaping microbial community structure, mediating nutrient cycling, and maintaining trophic dynamics in soil ecosystems^17^. Despite their ecological importance, research on the response of protists to PE exposure is still limited, and their interactions with bacterial and fungal communities within the plastisphere remain largely scarce. Moreover, it is essential to investigate the long-term legacy effects of PE exposure, as chronic accumulation may lead to persistent and systemic alterations in multi-kingdom microbiota that cannot be captured through short-term or single-domain studies^18^. Therefore, by incorporating bacteria, fungi, and protists and assessing their interactions under long-term field conditions, the current eight-year experiment provides an important advance over our earlier work, offering a more comprehensive understanding of plastisphere ecology and its broader environmental implications.

Another issue of particular concern is the dispersal of antibiotic resistance genes (ARGs) as a new emerging pollutant. The MPs can serve as a reservoir and refuge for ARGs and pathogenic bacteria, supporting their rapid spread due to their mobility, which further exacerbates the ecological risks^14^. The enrichment of high-risk ARGs in the plastisphere represents a serious ecological and public health concern, given their potential to spread through microbial networks and enter the human lymphatic systems, exacerbating the global issue of antibiotic resistance^19^. Hence, for enhancing public awareness of the potential human health risks associated with plastic pollution, further studies are urgently needed to evaluate the antibiotic resistome and other ecological threats. Previous research has highlighted the central role of bacterial community, particularly through their assembly processes, governed by the balance between deterministic and stochastic processes, in shaping ARGs dynamics through changes in bacterial composition, interactions, and niche selection^14,20,21^. However, the role of inter-kingdom interactions in ARGs dissemination remains largely unexplored. Associations between fungi, protists, and bacterial hosts may further influence ARGs spread through mechanisms such as co-colonization, resource competition, and microbial predation^22^. To date, only a few laboratory-or mesocosm-based studies have determined ARGs’ dissemination in the plastisphere, and these have been limited to a few types of ARGs^23^. Therefore, a comprehensive, field-based study that integrates multi-kingdom microbiota with ARGs profiling is essential for understanding the complex ecological drivers of ARGs propagation under plastic pollution.

To fill these knowledge gaps, we designed a long-term field experiment (eight years) integrating multi-omics and bioinformatics approaches to assess the ecological consequences of PE residues on multi-kingdom soil microbiota and the antibiotic resistome. Specifically, our study was guided by three hypotheses: (1) the soil microbiota is a complex, interdependent network of bacteria, fungi, and protists rather than isolated microbial domains; (2) this multi-kingdom community plays a critical role in shaping microbiota assembly and influences both biodegradation potential and ARGs dissemination within the plastisphere; and (3) protists, through trophic interactions such as predation, modulate inter-kingdom dynamics and ecosystem functioning, although their roles are often overlooked.

Accordingly, the specific objectives of this study were outlined as follows: (1) to characterize the diversity, composition, assembly processes, and functional profiles of multi-kingdom soil microbiota across bacteria, fungi, and protists under long-term PE exposure using amplicon and metagenomic sequencing, (2) to elucidate inter-kingdom microbial interactions through the integrated co-occurrence network analysis, and (3) to reveal the composition of ARGs and potential microbial pathogens and their associations with multi-kingdom microbiota using high-throughput qPCR techniques. Our findings aim to provide insights into the ecological drivers of plastisphere microbiota across multiple domains and underline the need to assess microbiome-mediated biodegradation and soil antibiotic resistome beyond a single microbial kingdom.

## Results

### Microbial diversity, composition, and assembly of multi-kingdom soil microbiota

**Fig. 1:**
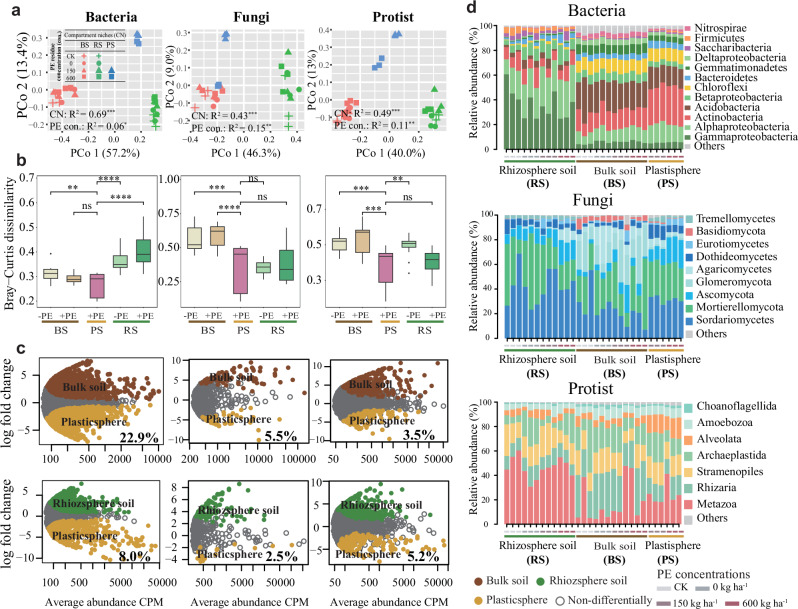
Microbial diversity and composition of bacterial, fungal, and protistan communities as influenced by different PE residue concentrations in different compartment niches (BS: bulk soil; RS: rhizosphere soil; PS: plastisphere). **a** Unconstrained PCoA ordinations based on the Bray–Curtis distance matrices of bacterial, fungal, and protistan communities as affected by different compartment niches and PE residue concentrations (0, 150, and 600 kg ha^–1^). **b** Bray–Curtis dissimilarities of bacterial, fungal, and protistan communities in response to PE residue (+PE) and without PE residue (−PE) in different compartment niches (*n* = 6). Black asterisks indicate significant difference (^*^p < 0.05; ^**^p < 0.01^; ***^p < 0.001; ^****^p < 0.0001), while “ns” represents non-significant differences between different treatments using the Wilcoxon rank-sum test. **c** Specific bacterial, fungal, and protistan species were enriched or depleted in the plastisphere compared to the bulk soil and rhizosphere compartments. *X*- and *Y*-axis represent the ASV abundance and log2-fold change (plastisphere compartment relative to bulk soil and rhizosphere compartments, respectively). Specific ASVs in the plastisphere, bulk soil, and rhizosphere compartments are colored in yellow, green, and brown, respectively. Gray-colored ASVs represent non-differentially abundant ASVs. The value in percentage inserted in each panel of Fig. 1c represents the proportion (%) of the number of specific ASVs enriched in the plastisphere relative to the total number of ASVs. **d** Taxonomic profiles of bacterial, fungal, and protistan composition at the phylum level as affected by different PE residue concentrations in different compartment niches. Phyla with relative abundances <1% were summarized within ‘Others’.

We first used amplicon sequencing to determine alpha- and beta-diversity and composition of multi-kingdom soil microbiota, and found that compartment niches explained a higher variation (43–69%) in comparison with PE residue concentrations (6–15%), through principal coordinates analysis (PCoA) ordinations and PERMANOVA analyses (Fig. 1a, Fig. S1). It was further observed that the contribution of PE residue concentration was higher (15% and 11%) in fungal and protistan communities compared to 6% in the bacterial community. The exogenous PE residues resulted in a greater disturbance in fungal and protistan communities, as is represented by Bray–Curtis distance matrices and dissimilarities, but less disturbance was evidenced in bacterial communities (Fig. 1b). This suggests that bacterial communities exhibit greater stability and adaptability to the plastisphere environment under PE exposure, compared to fungal and protistan communities. The stable absolute abundance of bacteria (log_10_ gene copies per gram soil), in contrast to the declines observed in fungi and protists, supports these findings (Fig. S2). Furthermore, this pattern was confirmed by the likelihood ratio test, revealing that the number of specific ASVs enriched in bacteria (22.9% and 8.0%) was much higher than that in fungi (5.5% and 2.5%) and protists (3.5% and 5.2%) in the plastisphere (Fig. 1c).

Taxonomic classification showed clear differences in the composition of all kingdoms at different compartmental niches and PE residue concentrations (Fig. 1d). The bacterial community was mainly composed of *Actinobacteria* (18.6%), *Alphaproteobacteria* (17%) and *Gammaproteobacteria* (16%); while *Sordariomycetes* (32%) and *Mortierellomycota* (30%) were the predominant phyla of fungal community, and *Metazoa* (30%) and *Rhizaria* (19%) for protistan community. Furthermore, at the highest concentration of PE residues (600 kg ha^–1^), several specific microbial phyla were significantly enriched in the plastisphere. Among bacteria, *Gammaproteobacteria*, *Gemmatimonadetes*, and *Firmicutes* increased by 11%, 7% and 56%, respectively (*p* < 0.05); *Ascomycota* increased by 47% among fungi, while *Amoebozoa* showed a 36% increase among protists (*p* < 0.05) (Fig. 1d; Table S1–S3).

**Fig. 2:**
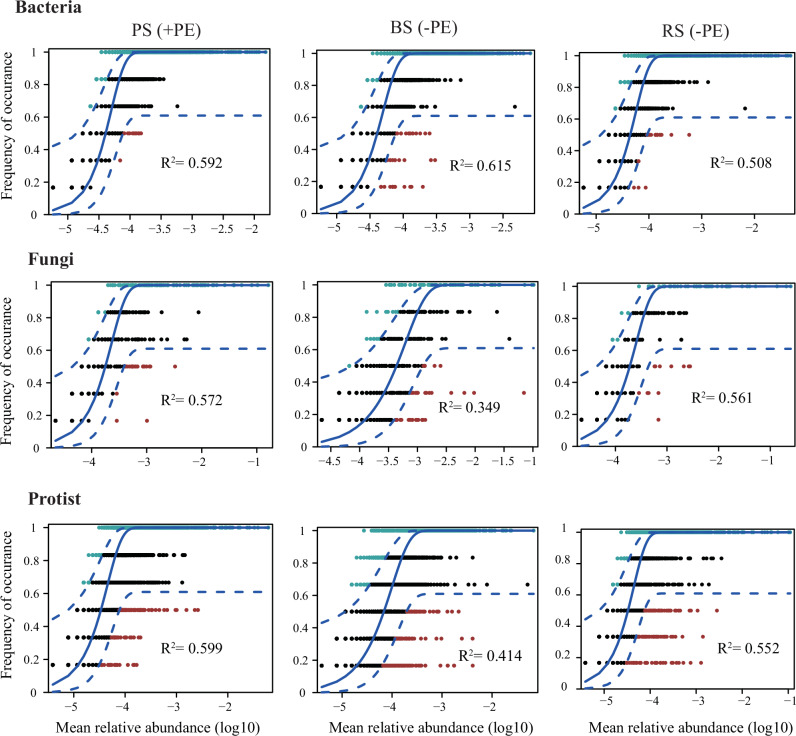
Fit of neutral model for bacterial, fungal, and protistan communities in different compartment niches (BS: bulk soil; RS: rhizosphere soil; PS: plastisphere). Each point represented a microbial ASV, and different colors indicated ASVs that occur more or less frequently than predicted by the neutral model. The predicted occurrence frequency was shown as a solid blue line, and dashed lines indicated a 95% confidence interval around the neutral model. The *R*^2^ indicated the fit to the neutral model. A higher *R*² indicates that stochastic processes play a greater role in shaping community assembly.

The neutral community model and null model were both employed to simulate the process of microbiota assembly between multi-kingdom domains. Most ASVs were distributed within the 95% confidence interval, with nearly 93%, 99%, and 98% of ASVs falling within the neutral model predictions for bacterial, fungal, and protistan communities, respectively (Fig. S3). Stochasticity processes in fungal and protistan communities (*R*^2^ = 0.572; *R*^2^ = 0.599) were higher under PE residue treatment compared to zero PE residues (*R*^2^ = 0.349; *R*^2^ = 0.414) (Fig. 2). These findings indicate that bacterial communities under PE exposure are primarily shaped by deterministic processes. This conclusion is further supported by βNTI values calculated using a null model with 999 randomizations (Fig. S4a), which consistently showed that deterministic processes dominate bacterial community assembly under PE exposure. Further analysis highlights that homogeneous selection plays a key role in driving this deterministic pattern (Fig. S4b). Collectively, these results suggest that environmental filtering, specifically imposed by PE exposure, deterministically structures bacterial community composition, in contrast to the more stochastic assembly observed under conditions without PE exposure.

### Inter-kingdom co-occurrence network and the associated topological characteristics

**Fig. 3:**
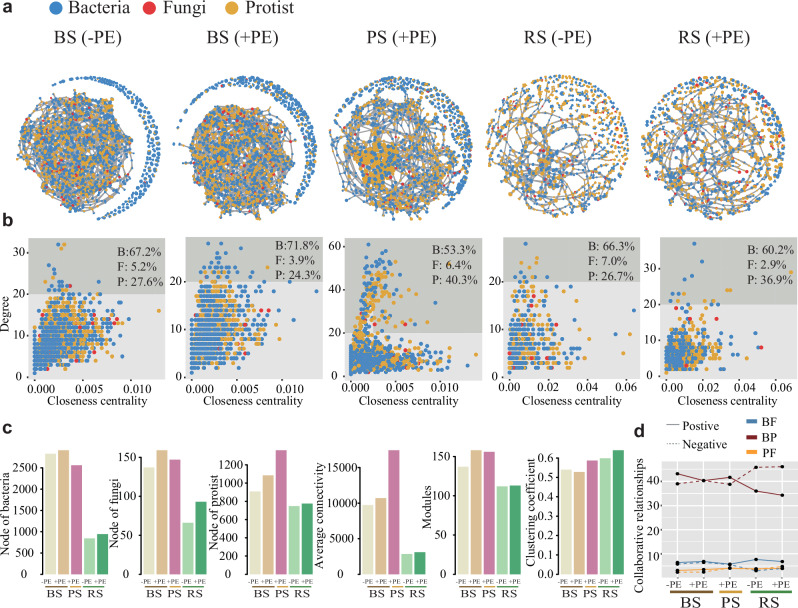
Interkingdom co-occurrence network between bacterial, fungal, and protistan communities and the associated characteristics in response to PE residue (+PE) and without PE residue (−PE) under different compartment niches (BS: bulk soil; RS: rhizosphere soil; PS: plastisphere). **a** Co-occurrence networks showing microbial interkingdom network patterns. **b** Biplot (degree and betweenness) of important ASVs (degreea 20). **c** Comparison of topological characteristics of interkingdom networks. **d** Positively and negatively collaborative relationships (represented by the proportion of edge, i.e., BF: bacteria-fungi edge; BP: bacteria-protist edge; PF: protist-fungi edge) between bacterial, fungal, and protistan taxa in the interkingdom network.

We further constructed inter-kingdom co-occurrence networks among bacterial, fungal, and protistan communities in response to PE residue applications to graphically visualize potential relationships between multi-kingdoms and their complexity (Fig. 3). Significant ASVs with a degree above 20 were further shown in a biplot (degree vs betweenness). These important ASVs mainly originated from the bacterial community, accounting for nearly 70% of the total, followed by protistan and fungal communities. Furthermore, the proportion of bacterial ASVs increased numerically with the application of PE residues compared to zero PE residues, especially in the plastisphere (Fig. 3b). However, the extent was relatively lower than that of fungi and protists, especially under PE residue application in the bulk soil and plastisphere compartment niches (Fig. S5). It can thus be inferred that those bacteria are the major constructors and play a predominant role in this inter-kingdom co-occurrence network.

The associated topological characteristics of inter-kingdom networks, such as nodes, average connectivity, modules, and clustering coefficients for bacteria, fungi, and protists, increased under the application of PE residues compared to zero PE residues (Fig. 3c). Positive and negative collaborative relationships between protists and bacteria, as indicated by the proportion of connection edges, were stronger in the plastisphere than in other combinations (Fig. 3d). Meanwhile, two specific protists of the *Sandonidae* genus, pASV1254 and pASV1722, were found to be closely correlated to other bacterial and fungal taxa (Fig. S6). These findings highlight the central role of the protist connector, revealed through cross-kingdom co-occurrence network analysis.

### The relationships of multi-kingdom soil microbiota with soil environmental factors

**Fig. 4:**
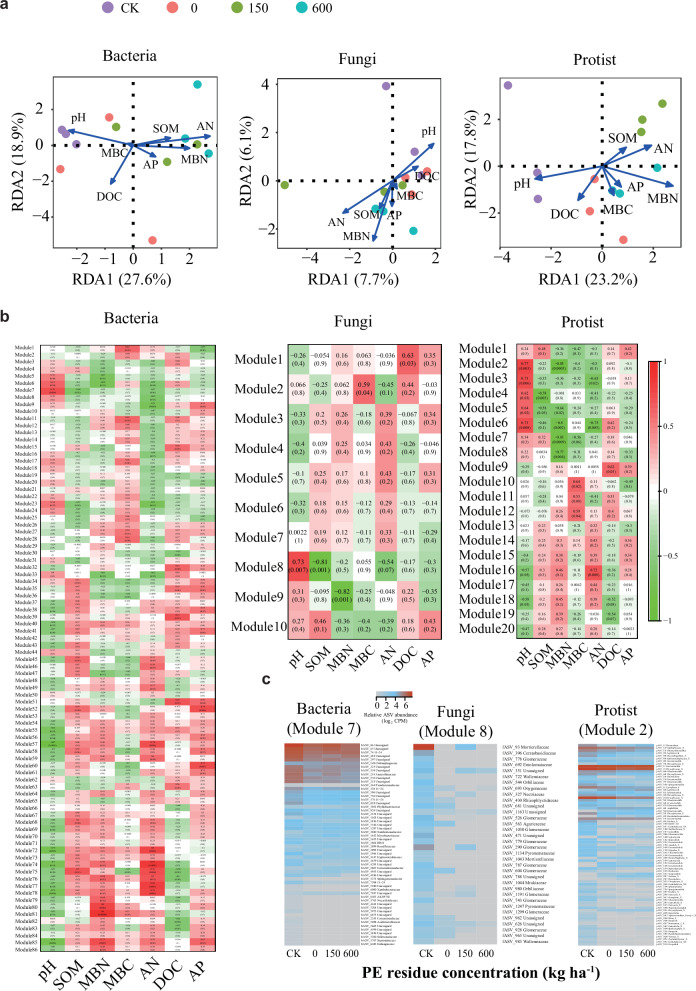
The relationships of bacterial, fungal, and protistan communities with several soil environmental factors. **a** Redundancy analysis of microbial community and soil environmental factors (SOM: soil organic matter; MBN: microbial biomass nitrogen; MBC: microbial biomass carbon; AN: available nitrogen; DOC: dissolved organic carbon; AP: available phosphorus). ^*^p < 0.1, ^**^p < 0.01, ^***^p < 0.001. **b** Microbial network modules were constructed by a weighted co-expression network with several soil environmental factors. **c** Heat map illustrating the relative abundance (counts per million; log_2_ scale) of ASVs belonging to the most relevant top module in association with pH, i.e., Modules 7, 8, and 2 in bacterial, fungal, and protistan communities, respectively, under different PE concentrations.

The concentration of applied PE residues significantly affected several soil environmental factors, such as AN, MBC, MBN, and pH (Table S4). In general, AN, AP, SOM, and MBN were enhanced with increasing concentration of PE residues. Furthermore, distance-based redundancy analysis (RDA) revealed that different PE residue concentrations clustered and were distinctly separated from each other. The highest rate of 600 kg ha^–1^ PE residues reduced soil pH (Fig. 4a).

The major microbial network modules were constructed through a weighted co-expression network, and then the relationships between several soil environmental factors and bacterial, fungal, and protistan communities were explored separately (Fig. 4b). The relative abundance of the shown ASVs belonged to the most relevant top modules related to pH, i.e., Modules 7, 8, and 2 in bacterial, fungal, and protistan communities, respectively (Fig. 4c). The abundance of these specific ASVs was relatively consistent within bacterial communities, but showed marked sensitivity in fungal and protistan communities when exposed to various PE residue concentrations (Fig. 4c). For example, the relative abundance of ASV in bacteria did not change with increasing concentrations of PE residues, whereas the relative abundance of fASV396, fASV778, and fASV692 in fungi was significantly decreased by nearly 80%. These results suggest that bacteria are more likely to adapt to abiotic stress environments, such as exogenous PE residues and the consequent reduction in pH, whereas fungi and protists are less adaptable.

### Functional profile of multi-kingdom microbiota through metagenomic analysis

**Fig. 5:**
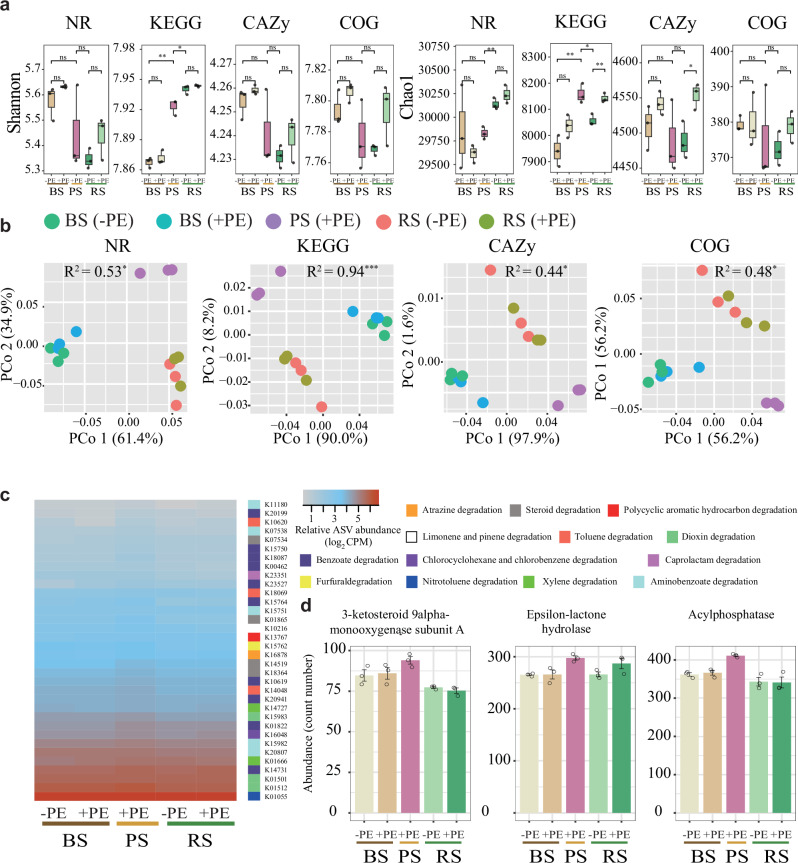
Microbial diversity and functional profiles derived from metagenome sequencing in response to PE residue (+PE) and without PE residue (−PE) under different compartment niches (BS: bulk soil; RS: rhizosphere soil; PS: plastisphere). **a** Alpha diversity indices (Shannon and Chao1) of KO, CAZyome, COG, and NR functional genes (*n* = 3). Black asterisks indicate significant difference (^*^p < 0.05; ^**^p < 0.01^; ***^p < 0.001), while “ns” represents non-significant differences (Wilcoxon rank-sum test). **b** Principal coordinate analysis (PCoA) plots based on Bray–Curtis distance matrices of KO, CAZyome, COG, and NR functional genes. **c** Heat map of the relative abundance (Z-score) of KO functional genes involved with xenobiotics biodegradation and metabolism pathways. **d** Abundance (read counts) of three KOs functional genes (*K15983*, *K14731*, and *K01512*). Error bars represent mean ± standard error (SE), with each data point indicating an individual sample library (*n*  =  3).

Metagenomic analysis revealed that functional genes of the plastisphere were found to be far away from those of other compartment niches, regardless of whether or not they had PE residues. Microbiome functional diversities (Chao1 and Shannon indexes) were generally higher under the application of PE residues, compared to zero PE residues, regardless of different compartment niches (*p* < 0.05; Fig. 5a). In particular, the microbiome functional genes (KO, CAZyome, COG, and NR), beyond taxonomic composition, and the data revealed that different groups were clustered and distinctly separated from each other, through PCoA ordinations and PERMANOVA analyses (*R*^2^ = 0.44^*^–0.94^***^; Fig. 5b).

Specific microbiome functional genes enriched at the plastisphere, compared to other compartment niches, in the function of xenobiotic biodegradation were selected and shown in Fig. 5c. Notably, the abundance of functional genes K15983, K14731, and K01512 under the plastisphere was higher than in other compartment niches (Fig. 5d). Meanwhile, the processes of xenobiotic biodegradation mainly involve the degradation of atrazine, chloroalkane, toluene, chloroalkene, chlorocyclohexane and chlorobenzene. Herein, a random forest regression model was employed to predict functional genes in the KEGG database related to xenobiotic biodegradation and metabolic pathways, as driven by different inter-kingdom microbiomes. The results revealed that bacterial communities were the major contributor to predicted functional genera, followed by protistan and fungal communities (Fig. S7a). Furthermore, a set of 56 bacterial biomarkers was identified based on minimal CV and ranked according to their relative importance (Fig. S7b, c). Their phylogenetic relationship was also reconstructed to reveal taxonomic patterns (Fig. S7d). In addition, several KO functional genes, such as hydrolases (*E3.1.1.45*, *amiE,* and *URA4*) and oxidoreductases (*sdhB*, *mdh,* and *frmA*), were found to be significantly enriched in the plastisphere compared to other soil compartments (*p* < 0.05), indicating a potential enhancement of microbial functional capacity related to carbon degradation and redox processes (Fig. S8). Several functional genes related to C, N, and S cycling that responded significantly to the application of PE residues were selected using Linear discriminant analysis (LDA) effect size (LEfSe) (Fig. S9). These selected functional genes exhibited the highest abundance in the plastisphere, compared to other compartmental niches, and in most cases, this abundance also increased significantly with increasing concentration of PE residues (Table S5; Fig. S10). The abundance of arsenic resistance genes (*arsB* and *arsH*) in the KEGG database increased greatly with the application of PE residues compared to zero PE residues in the bulk and rhizosphere soil compartments (Fig. S11). The responses of potential pathogens, as identified through amplicon sequencing, and the most abundant functional genes annotated by the virulence factor database (VFDB), bacterial metal resistance genes database (BacMet), and pathogen–host interactions database (PHI), to PE residues exhibited an almost similar trend (Figs. S12–13).

### ARGs and their relationship with microbiome assembly

High-throughput qPCR detection of ARGs was conducted to gain an in-depth understanding of the antibiotic resistome, using the SmartChip Real-Time PCR System. A total of 184 ARGs belonging to ten types and 38 mobile genetic elements (MGEs) were detected in this study. PCoA ordination of ARGs profiles showed that different compartment niches were distinctly separated from each other, which explained nearly 63% of the variation (Fig. S14a). The top two dominant ARGs types were Aminoglycoside antibiotic and Multidrug, which were significantly higher under the application of PE residues, compared to no PE residues, regardless of compartment niches (Fig. S14b). This result was further supported by the observation that the cumulative abundance of ARGs, annotated using the Comprehensive Antibiotic Resistance Database (CARD) through metagenomic sequencing, increased greatly under PE residue compared to the no PE residues (Fig. S13).

**Fig. 6:**
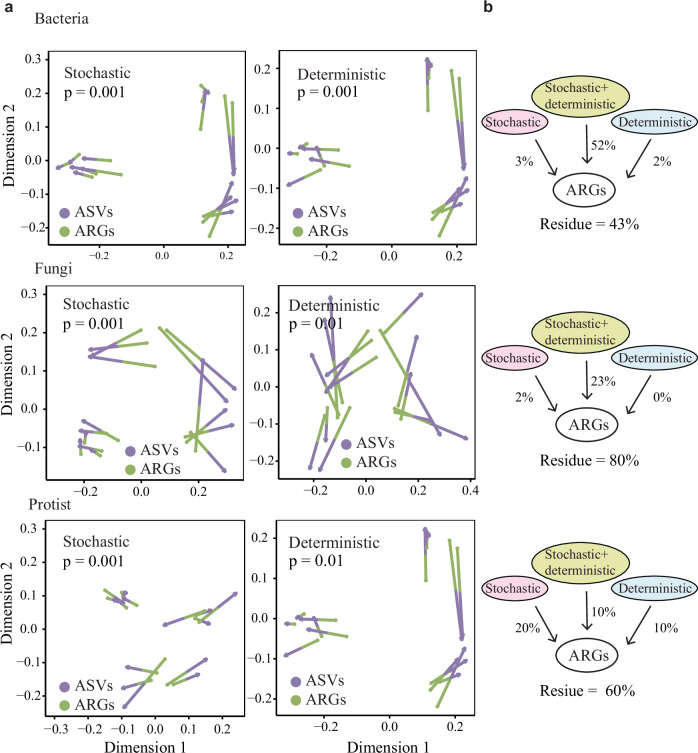
The relationships between microbial assembly processes (deterministic and stochastic processes) and ARG profiles. **a** Correlations between ARG profiles and microbial communities from deterministic and stochastic processes using the Procrustes test. **b** The contribution of deterministic and stochastic processes on ARGs profiles in bacterial, fungal, and protistan communities using the variance partitioning analysis.

Procrustes analysis showed a strong relationship (*P* < 0.01) between microbiome assembly processes (deterministic and stochastic processes) and ARGs profiles (Fig. 6a). Variance partitioning analysis was further applied to determine the relative contributions of deterministic and stochastic processes in ARGs profiles. Interestingly, bacterial assembly (stochastic plus deterministic) processes contributed the most to the ARGs profiles, exhibiting an approximate value of nearly 52%, while fungal and protist assemblies contributed only 23% and 10%, respectively (Fig. 6b). We further constructed co-occurrence networks to examine the relationships between ARGs and multi-kingdom microbiota in response to PE exposure. The results showed that key topological features of the network, including the number of connected edges between bacterial hosts and ARGs, as well as overall network density and centralization, increased substantially under PE exposure compared to the control (Fig. S15a, b). In addition, fungal and protistan genera exhibited positive correlations with bacterial hosts within the inter-kingdom network (Fig. S15c, d). The effect of PE residues on ARG abundance, mediated through inter-kingdom microbial communities and soil pH, was evaluated using a structural equation model (SEM), which highlighted the predominant role of the bacterial community in shaping ARG dissemination (Fig. S16).

## Discussion

As was hypothesized, our data demonstrate that soil microbiota are not simply a collection of independent domains/populations, but constitute a diverse and complex community whose members coexist and interact physically in most natural environments^24^. Because of such inter-kingdom interactions, multi-kingdom communities often have functions that are different from the superposition of each single kingdom, and may execute a crucial role in microbiota assembly and biodegradation functioning^25,26^. We therefore sought to consider this research gap and adopted a more comprehensive approach for holistic insights into their multi-kingdom relationships. The present study clearly showed that bacteria have an inherent advantage owing to their easier adaptation to conditions of PE exposure and can thus play a major role in degradation functioning and ARG dissemination, compared to the other two kingdoms^27^. Further discussion on such inter-kingdom variations is given below.

Firstly, the evidence of beta-diversity and composition of multi-kingdom soil microbiota suggested that the bacterial communities are more stable and better adapted to survive in the adverse environment when exposed to PE residues than fungal and protistan communities. Such results were further authenticated by qPCR and likelihood ratio tests, revealing a larger number of specific ASVs enriched in the plastisphere for bacteria than both fungi and protists in this study (Fig. S2; Fig. 1c). These observations are consistent with previous studies, showing that MP exposure reduced the stability of fungal communities by weakening symbiotic relationships between intra-kingdom species^28,29^. Conversely, bacteria exhibit synergistic living habits in response to MP exposure; for example, some specific bacterial species, such as *hyphomonadaceae*, can produce a polysaccharide holdfast that enables them to attach firmly to plastisphere^30^. Similarly, *erythrobacteraceae* can degrade polycyclic aromatic hydrocarbons via hydrolase enzymes, which makes these species prime candidates for colonizing in plastisphere^31^.

Secondly, both the neutral community model and the null model were applied to simulate the microbiota assembly processes. Results from both approaches indicated that deterministic processes dominated community assembly for bacteria, whereas fungal and protistan communities were more influenced by stochastic processes under PE exposure. Further analysis revealed that homogeneous selection plays a central role in driving this deterministic pattern for the bacterial community (Fig. 2; Fig. S4). This may be attributed to strong environmental filtering imposed by PE residues, which create relatively uniform physicochemical conditions, such as local redox microenvironments that selectively favor bacterial taxa with specific physiological and metabolic traits^29^. Under such a situation, some bacteria capable of utilizing plastic-derived compounds, forming biofilms on hydrophobic surfaces, or tolerating oxidative stress may gain a competitive advantage^30^. The resultant selective pressures reduce the influence of stochastic colonization and promote the proliferation of functionally similar taxa, leading to community convergence under PE exposure, an outcome consistent with deterministic theory in community ecology^15^. Indeed, the shift in microbiota assembly appears to enhance the community’s biodegradation potential, as evidenced by the increased abundance of several C-cycling functional genes, which are discussed in detail below.

A noteworthy aspect of this study is that the analysis of inter-kingdom co-occurrence network strengthens the importance of bacteria, which implies that bacteria are the main constructors in network construction. Of note, protists have been identified as major connectors in inter-kingdom co-occurrence networks, specifically two species of pASV1254 and pASV1722 belonging to the bacterivorous genus *Sandonidae*, due to their close relationships with bacterial and fungal communities (Fig. S6). The existing literature also suggests that protists have distinct ecological mechanisms, such as generalist predation on any bacterial and fungal species, or mutualistic, facilitative, predatory, and parasitic interactions between different taxa across different inter-kingdom domains^32,33^. This can certainly help protists to better cooperate with both bacteria and fungi to resist adverse environments via molecules and induction of signaling pathways, and compete with or complement each other concerning nutrient acquisition, metabolism, and growth, upon exposure to PE residues^34^. Such synergetic interactions among various multi-kingdom domains will facilitate the execution of biodegradation functions. This also fits well with the stress gradient hypothesis that strong interactions tend to dominate over competitive interactions in “harsher” environments, as is found in terrestrial ecosystems^14,35^.

This study also highlights the decrease in soil pH at higher concentrations of PE residues. Previous studies have indicated that during plastic biodegradation, large amounts of dissolved organic carbon and other chemical compounds are often released, which significantly reduce soil pH^36,37^. We further identified the main microbial network modules through a weighted co-expression network and then figured out the specific ASVs belonging to the most relevant top modules related to pH (Fig. 4). Notably, bacterial ASVs were relatively unaltered, whereas fungal and protistan ASVs were sensitive when exposed to increasing concentrations of PE residues. These results suggest that bacteria are more likely to adapt to abiotic stress environments, such as exogenous PE residues and consequent pH reduction (Fig. S16), whereas fungi and protists are less adaptable. Collectively, these findings demonstrate that bacteria have higher adaptability and play a more important role in ecological processes than fungi and protists. Likewise, protists act as key bridges linking bacterial and fungal communities when exposed to adverse environmental conditions (e.g., PE residues).

In line with existing literature, the current study, through metagenomics sequencing, revealed that PE residues increased the functional diversity of microbiome^38,39^. Specifically, consistent with previous studies^14,28^ several metabolic pathways associated with xenobiotic biodegradation, as well as functional genes encoding hydrolases and oxidoreductases, were concurrently enriched in the plastisphere (Fig. 5; Fig. S8). Notably, several key functional genes identified in this study, such as *sdhA*, *SDHB*, and *pht5*, have been previously reported to play essential roles in the TCA cycle and the degradation of polycyclic aromatic hydrocarbons^40–42^. Furthermore, using a random forest model, we found that bacterial ASVs were the major contributor to predicted functional genes, followed by protistan and fungal ASVs, further emphasizing the dominant role of bacteria across multi-kingdom domains. Specifically, our study identified some key taxa (ASV6, ASV20, ASV1140, ASV1264, and ASV1197) from two families (*Pseudonocardiaceae* and *Xanthomonadaceae*) that were previously reported to be responsible for the biodegradation of poly(lactic) acid and pentachlorophenol^43,44^. In addition, several functional genes involved in C-, N-, and S-cycling that responded significantly to PE exposure were identified using LEfSe analysis. Specifically, C-cycling genes related to C degradation, fixation, and metabolism, along with N-cycling genes involved in ammonia assimilation, assimilatory nitrate reduction, and denitrification, were notably enriched in the plastisphere (Fig. S10). For instance, the *ppdK* gene, previously reported to play a key role in glycolysis^44^, exhibited relatively higher abundance in the plastisphere. The enrichment of C- and N-cycling genes under PE exposure indicates a functional enhancement of microbial processes related to nutrient cycling, potentially improving the availability of nitrogen and carbon to plants^45^.

In order to raise public awareness of plastic pollution, an in-depth understanding of the antibiotic resistome in response to PE exposure and its influencing factors is required. Our study found the presence of a range of ARGs in the plastisphere as “hot spots”, suggesting an increased risk of ARGs spreading and prevalence through MGEs. Several recent studies have also detected many unique ARGs and MGEs in the plastisphere, which were significantly higher than those in the soil^14^. Exchange of ARGs may frequently occur in some plastispheres from the soil ecosystem via MGEs, as a horizontal gene transfer^3^. Multidrug resistance genes are an important category of ARG types, whose abundance was detected to increase at higher PE residue concentrations. A probable explanation is that multiple pollutants were absorbed by the microbiota when subjected to higher concentrations of PE residues^46^. This assumption was also partly supported by the increase in arsenic resistance genes (*arsB* and *arsH*) in the KEGG database upon exposure to PE residues (Fig. S11). Existing literature also highlights that metabolic pathways belonging to pollutants were enriched in the plastisphere through metagenomic sequencing^47,48^. In addition, we also found that potential pathogens through the amplicon sequencing and the virulence factors annotated by the VFDB were enriched in the plastisphere. All these findings suggest that PE residues can act as reservoirs and refugia that accelerate the dispersal of ARGs and new pollutants in terrestrial ecosystems^14^.

A significant correlation has been previously evidenced between bacterial community and ARG profiles^46^. Our study revealed, by Procrustes test and variance partitioning analysis, that bacterial assembly (stochastic plus deterministic) processes were the largest contributors to antibiotic resistome in plastispheres. This implies that bacterial assembly in the plastisphere under PE exposure can play a critical role in the enrichment of ARGs. We further constructed co-occurrence networks to explore the relationships between ARGs and multi-kingdom microbiota in response to PE exposure (Fig. S15). The increased connectivity between bacterial hosts and ARGs under PE exposure reflects the central role of bacterial hosts in driving ARG dissemination, which can be attributed to selective pressures arising from the altered microenvironment. Notably, the involvement of fungal and protistan genera in the inter-kingdom network, evidenced by their positive correlations with bacterial hosts, further implies that ARGs dissemination is shaped not only by bacterial hosts but also by broader ecological interactions across microbial kingdoms. These findings were further supported by SEM analysis (Fig. S16) and underscore the importance of considering multi-kingdom dynamics when evaluating the spread of ARGs in environments impacted by plastic pollution.

Over the eight years of continuous field experimentation in this study, the PE residues were likely subjected to mechanical fragmentation, macrobiological disturbance, and chemical weathering, processes known to gradually modify their morphology and surface properties, even though we did not directly quantify these changes^49^. Mechanical forces such as tillage and soil abrasion, along with macrofaunal activities (e.g., ingestion, chewing, and excretion by earthworms or insects), can produce cracks, grooves, and increased surface roughness^50^. In parallel, abiotic and chemical weathering processes (such as photo-oxidation, hydrolysis, and thermal cycling) introduce carbonyl and hydroxyl groups, which reduce molecular weight and alter crystallinity^50,51^. These transformations increase the specific surface area and heterogeneity of PE residues, thereby enhancing microbial colonization and reshaping community assembly on plastic surfaces. They also generate micro-niches that facilitate microbial adhesion, biofilm development, and the recruitment of diverse microbial taxa. Consequently, long-term aging of PE residues may drive shifts in plastisphere diversity and function, potentially enriching microbial groups involved in hydrocarbon degradation, redox transformations, and ARG dissemination, as evidenced in our results and discussed above.

In summary, the scientific evidence from the holistic investigation of multi-kingdom microbiota presented in this study provides innovative insights into soil microbiota assembly and ecological interactions across multiple realms. It also underlines the importance of a continued research effort to understand the biodegradation of microbiome-mediated PE residues beyond a single microbial kingdom. These results have far-reaching implications for evaluating the impact of PE residues in farmland soils, which are pivotal for global food security and environmental sustainability.

## Methods

### Field preparation and experimental design

Field experiments were executed from 2014 to 2021 at the Changwu Agro-Ecological Research Station located in the Loess Plateau of Shaanxi province, northwest China (107°40 E, 35°12 N). Maize was planted during April and harvested in September each year, then the field was left fallow until April of the following year. The experimental site was characterized by a dry semi-humid climate. The annual average precipitation and air temperature from 2020 to 2021 were 584.1 mm and 10.1 ^o^C, respectively. The experimental field had dark loessal soil. The topsoil (0–20 cm) in the sampling year of 2021 contained 14.7 g kg^–1^ soil organic matter, 2.70 mg kg^–1^ available N, 7.33 mg kg^–1^ available P, with a pH of 7.93. Each plot measured 4 m × 4 m, with a 1-meter-wide isolation zone between plots to ensure the independence of treatments.

The treatments were established in the year 2014 by installing three different PE residue concentrations (0, 150, and 600 kg PE ha^–1^) under plastic film mulching conditions. These three concentrations were selected to represent the current ‘ambient exposure’, ‘moderate exposure’, and ‘maximum exposure’ of Chinese farmland^52,53^. These selected PE residue concentrations are within the environmental occurrence range and could be regarded as the realistic doses under film-covered field conditions in Chinese farmland. Meanwhile, an additional treatment of zero PE concentration and without plastic film mulching was used as the control in the field experiment. The PE residue fragments were only applied in the starting year of 2014 and retained in each plot for long-term observation until 2021. The field experiments were performed according to a randomized complete block design with three replications.

The field was tilled before mulching, when a basal dose of N (180 kg N ha^–1^) and P (40 kg P ha^–1^) fertilizer, was spread over the topsoil and, followed by the weighing and spreading of the PE residue fragment on the soil surface. The PE residues were incorporated manually into the topsoil layer (0–30 cm) in each treatment. The PE residue fragments (0.008 mm-thick) were obtained by cutting the plastic film into small pieces (4 × 5 cm) with scissors. After the PE residual debris was incorporated into the topsoil, ridges and furrows were formed artificially with a width of 55 cm. Then, the alternate ridges were mulched using the PE film. After maize harvesting, the mulching films were consistently removed at the end of each cropping season. This procedure was applied uniformly across all treatment plots to minimize potential bias from mulching film residues as a confounding factor.

### Samples collection, measurements and amplicon sequencing

Soil samples of the three-compartment niches were collected during the maize mature stage for all the plots in 2021. Bulk soil samples were collected from the topsoil of corn plants between two rows using a stainless-steel auger with a 3 cm diameter. Five representative subsamples in the bulk soil were mixed thoroughly, passed through a 2 mm steel mesh, and collected as a sample for each plot. Rhizosphere compartments that were tightly attached to the fine roots were also sampled for each plot. For the plastisphere samples, the PE residues were collected artificially from the topsoil, and the soil sample tightly bound to the film surface was extracted using the following method. First, it was immersed in a sterile saline solution, and the soil suspension was centrifuged at 10000 ×g for 10 min; the resulting pellet was defined as plastisphere soil. The bulk soil and rhizosphere samples were passed through a 2 mm steel mesh to remove the roots and debris. All samples were immediately sealed in sterile polyethylene bags and transported to the laboratory within 6 hours in a portable refrigerator containing ice. The bulk soil and rhizosphere samples were partitioned into two parts. One subsample was stored at 4 °C to measure soil chemical properties. Another subsample and the plastisphere samples were stored at -80 °C for DNA extraction. Plastisphere samples were not used for soil chemical property measurements because the amount was too small and was only stored at -80 °C for DNA extraction.

Before the measurement of soil chemical properties, soil moisture was recorded using the oven-drying method. Soil pH (1:1 soil/water, w/w) was determined directly after shaking for 1 h using a digital pH meter (STEH-200N, Shanghai, China). Available N (NH_4_^+^ and NO_3_^–^) in the soil was extracted with 1 M KCl solution, and its concentration in the extract was analyzed using a standard colourimetric procedure with an automatic discrete analyzer (DeChem-Tech, Hamburg, Germany)^54^. Available P was determined using the sodium bicarbonate extraction method^55^. Dissolved organic carbon (DOC, mg kg^–1^) was extracted using the same solution as inorganic N and analyzed using a total organic carbon analyzer (TOC-L CPH, Shimadzu Corp. Japan). Microbial biomass carbon (MBC, mg kg^–1^), microbial biomass nitrogen (MBN, mg kg^–1^) and microbial biomass phosphorus (MBP, mg kg^–1^) were measured using the chloroform-fumigation extraction technique^56–58^.

Soil genomic DNA was extracted from 0.5 g of fresh soil using FastDNA^TM^ SPIN Kit for Soil (MP Biomedicals, Santa Ana, USA) and stored at −20 °C prior to further analysis. The quality of the DNA extract was checked on a 1% agarose gel, and DNA concentration and purity were determined using a NanoDrop 2000 UV vis spectrophotometer (Thermo Scientific, Wilmington, USA). The V3-V4 hyper-variable region of the 16S rRNA gene was amplified with the primer pair 338 F (5’-ACTCCTACGGGAGGCAGCAG-3’) and 806 R (5’-GGACTACHVGGGTWTCTAAT-3’); fungal ITS1-2 region was amplified using primers ITS1(5’-TCCGTAGGTGAACCTGCGG-3’) and ITS2(5’- GCTGCGTTCTTCATCGATGC-3’); 18S V9 hypervariable region of eukaryotes was amplified using primers TAReuk454FWD1 (5’-CCAGCASCYGCGGTAATTCC-3’) and TAReukREV3 (5’-ACTTTCGTTCTTGATYRA-3’), using a PCR thermo-cycler (Applied Biosystems ABI 9700, CA, USA). The PCR product was extracted from a 2% agarose gel, purified using AxyPrep DNA Gel Extraction Kit (Axygen Biosciences, Union City, CA, USA) according to the manufacturer’s instructions, and quantified using a Quantus™ Fluorometer (Promega, USA). Purified amplicons were pooled in equimolar and paired-end sequences on an Illumina MiSeq PE300 platform (Illumina, San Diego, USA) according to the standard protocol.

### Bioinformatics analysis

Raw sequencing reads were pre-quality filtered and trimmed using fastp version 0.20.0^59^ and merged using FLASH^60^. Sequences were processed using USEARCH 10.0 software and VSEARCH 2.14 software^61,62^ and amplicon sequence variants (ASVs) were identified using the DADA2 algorithm^63^. Bacterial, fungal, and protistan taxonomic assignments were performed using SILVA (v13.8), UNITE (v8.0), and PR2 (v4.12.0) reference databases, respectively. A total of 1,033,539 bacterial, 892,189 fungal, and 2,330,351 protist high-quality reads were retrieved from 30 samples and sorted into 7178 bacterial, 976 fungal, and 3645 protistan ASVs. The bacterial, fungal, and protist ASV tables were then rarefied to 28,796, 7,574, and 42,744 reads (the lowest number among all samples), respectively, for comparison on an equal basis for general analysis calculations.

### Quantitative PCR for targeting specific genes

The absolute abundances of bacteria (16S rRNA genes), fungi (ITS region), and protists (18S rRNA genes) were quantified using quantitative PCR (qPCR) with universal primer sets: 338F (5’-ACTCCTACGGGAGGCAGCAG-3’)/806R (5’-GGACTACHVGGGTWTCTAAT-3’) for bacteria, ITSF3 (5’-GCATCGATGAAGAACGCAGC-3’)/ITSF4 (5’-TCCTCCGCTTATTGATATGC-3’) for fungi, and 528F (5’-GCGGTAATTCCAGCTCCAA-3’)/706R (5’-AATCCRAGAATTTCACCTCT-3’) for protists. The qPCR procedures followed the protocol described by Fierer et al^64^. Quantification was achieved using gene-specific standard curves, and results were expressed as log10-transformed gene copy numbers per gram of dry soil.

### High-throughput qPCR for detection of ARGs

High-throughput qPCR is a highly specialized and sensitive method that enables absolute quantification of ARGs with high accuracy and reproducibility. A total of 184 ARGs, 38 MGEs, and the 16S rRNA genes were determined using this method. Primers and PCR conditions for gene amplifications were as described in previous studies^65^. The qPCR reaction and fluorescence signal detection were performed in the SmartChip Real-Time PCR system (WaferGen Biosystems USA), and the amplification curve and dissolution curve were generated. Data with low amplification efficiencies (<1.8) or multiple melting peaks were removed, and the detection limit of amplification was set to threshold cycle (CT) of 31. Technically, amplification of each DNA sample was replicated three times. SmartChip qPCR software was used to process the raw output data. This study used the relative abundance of ARGs, calculated according to previously reported equations^65^. The Ct values of each gene were produced by the SmartChip Real-Time PCR System (WaferGen Biosystems USA). The gene will be discarded (1) when the amplification efficiency was less than 1.8 or greater than 2.2; (2) the negative control was amplified; (3) the Ct value was greater than 31. Only genes detected by three technical repetitions can be judged positive. In this study, 15 samples were selected, including three compartment niches and two PE residue concentrations (0 and 600 kg ha^–1^) for high-throughput detection of ARGs.

### Metagenomic sequencing and data mining

Metagenomic sequencing was conducted for further microbial function profiles using the Illumina NovaSeq platform with paired-end protocols. Sequencing libraries and index codes were generated using the ALFA-SEQ DNA Library Prep Kit following the manufacturer’s recommendations. The library quality was assessed using the Qubit 4.0 Fluorometer (Life Technologies, Grand Island, NY) and Qsep400 High-Throughput Nucleic Acid Protein Analysis (Guangdong Magigene Biotechnology Co., Ltd., Guangzhou, China) system. At last, the library was sequenced on an Illumina NovaSeq 6000 platform, and 150 bp paired-end reads were generated. The raw data were processed using Trimmomatic (v.0.39)^66^ to acquire the clean data for further analysis. An average of 2 Gb of clean data was retrieved for each sample.

The reads of all samples were combined and then used the software MEGAHIT (Version v1.0.6)^67^ for mixed assembly with the same parameters as single assembly. Filter the fragment shorter than 500 bp in all of Scaftigs for statistical analysis and predicted the ORF by MetaGeneMark (Version 3.38), and filtered the length information shorter than 90 nt from the predicted result with default parameters. The CD-HIT (Version: 4.7) was adopted to remove redundancy and obtain the unique initial gene catalog, which was clustered by identity 95%, coverage 90%, and chose the longest representative sequences. The clean data of each sample were mapped to the initial gene catalog using BBMAP software, and the number of reads mapped to genes in each sample was obtained. Based on the number of mapped reads and the length of the gene, the abundance information of each gene in each sample was calculated.

DIAMOND software was used to blast the unigenes against the sequences of bacteria, fungi, archaea, and viruses, which were all extracted from the NR database of NCBI. MEGAN software was used to ensure the species annotation information of sequences. Functional database included KEGG database, eggnog, and CAZy database. For each sequence’s blast result, the best Blast Hit is used for subsequent analysis. We also used metagenomic sequencing to detect ARGs by aligning clean reads against the Comprehensive Antibiotic Resistance Database (CARD), as it provides broad coverage and valuable insights into their genetic context. By integrating metagenomics with high-throughput qPCR, we aimed to enhance cross-validation results and ensure robust quantification of ARGs. In this metagenomic analysis, 15 samples were selected across three compartment niches (bulk soil, rhizosphere soil, and plastisphere) and two PE residue concentrations (0 and 600 kg ha⁻¹).

### Statistics and reproducibility

PE residues had been continuously immersed in the topsoil for eight consecutive years, enabling us to evaluate their legacy effect. In 2021, soil samples were collected at the maize maturity stage from three different compartment niches (bulk soil, rhizosphere soil, and plastisphere). In total, 30 soil samples were taken and stored at −80 °C before DNA extraction. For amplicon sequencing, all 30 DNA samples were analyzed. For metagenome sequencing and high-throughput qPCR for detection of ARGs, a subset of 15 samples was selected. These represented three-compartment niches under two PE residue concentrations, with three replicates for each treatment−compartment combination.

Alpha-diversity was calculated using the “*diversity*” and “*estimate*” function in “*vegan*” package of R^68^. Taxonomic differences, soil chemical properties, and functional genes (count no.) of different treatments were tested using Tukey’s post hoc test at a 95% confidence interval using the “*HSD.test*” function in *agricolae* package of R^69^. All other comparisons were examined in Wilcoxon rank-sum tests, using the “*stat_compare_means*” function in “*ggplot2*” package of R^70^. The beta-diversity was ordinated in weighted Bray–Curtis distance matrices using unconstrained PCoA, and then plotted in the *phyloseq* package of R for bacterial, fungal, and protistan communities, separately^71^. The significant difference among various influencing factors on the dissimilarity of bacterial, fungal, and protistan communities was tested with PERMANOVA using “*Adonis*” function of the R package “*vegan*”^68^. The differential ASVs for bacterial, fungal, and protistan communities in bulk soil, rhizosphere soil, and plastisphere niches were detected using likelihood ratio tests and false discovery rate corrected value of p < 0.05 with the R package *edgeR*^72^. SEM was constructed using the “*lavaan*” package in R software to assess the influence of PE residues on soil pH, inter-kingdom microbial communities, and ARG abundance^73^.

To assess the relative contributions of deterministic and stochastic processes in microbiome assembly, the beta Nearest Taxon Index (βNTI) was calculated using a null model with 999 randomizations^74^. Values of |βNTI| ≥ 2 were interpreted as evidence of deterministic processes, whereas |βNTI| < 2 indicated stochastic processes as dominant drivers^75,76^. Based on the combination of βNTI and Bray–Curtis–based Raup–Crick Index (RCBray) values, these ecological processes were partitioned into five categories: heterogeneous selection (βNTI < –2), homogeneous selection (βNTI > +2), dispersal limitation (|βNTI| < 2 and RCBray > 0.95), homogenizing dispersal (|βNTI| < 2 and RCBray < –0.95), and undominated processes (|βNTI| < 2 and |RCBray| < 0.95)^75,76^. In parallel, a neutral community model was constructed to further examine the role of stochasticity in community assembly for bacterial, fungal, and protistan communities, separately. This approach follows the framework described by Sloan et al.^77^, based on three key assumptions: (1) ASVs were selected randomly from a common regional species pool; (2) the death rate of microbial community was equivalent to the growth rate; and (3) the dispersal efficiency of ASVs was comparable for different compartment niches.

Inter-kingdom and intra-kingdom co-occurrence networks among bacterial, fungal, and protistan communities were constructed based on Spearman rank correlation coefficients (Spearman’s ρ > 0.7 or ρ < −0.7; P < 0.001) using all ASVs across the three domains. Additionally, to investigate the relationships between bacteria (as potential hosts) and ARGs, we constructed co-occurrence networks between these two groups using the same correlation thresholds (Spearman’s ρ > 0.7; *P* < 0.001). The bacterial genera identified as potential ARG hosts were then integrated into a multi-kingdom network alongside fungal and protistan communities to further explore inter-domain ecological interactions. The networks were visualized by the layout of Fruchterman–Reingold with 10^4^ permutations in the R package “*igraph*” and Cytoscape 3.9.1 software^78^. Network topological characteristics, consisting of the number of nodes, average connectivity, number of modules, clustering coefficient, and degree and closeness centrality, were calculated using the *“igraph”*. These topological characteristics were used to quantify the connectivity and complexity of co-occurrence networks across the inter-kingdom^78^. It should be noted that the co-occurrence networks presented in this study reflect correlation-based associations rather than direct causal relationships between microbial taxa or ARGs.

RDA based on the Bray–Curtis dissimilarity metric was conducted to determine the influence of soil chemical properties on bacterial, fungal, and protistan communities, using the R package “*vegan*”^68^. A weighted co-expression network analysis was constructed to identify key modules of highly correlated ASVs for bacterial, fungal, and protistan communities, separately, using the R package “*WGCNA*”^79^. The soft-thresholding power of the correlation network was set to four, and the minimum module size was set to 30. The Spearman’s correlations of these key modules of ASVs and soil environmental factors, and then the important ASVs that contributed to the specific module affecting pH, were illustrated in heatmaps.

LDA effect size (LEfSe) was run using the R package “*microeco*” to identify functional genes in the KO database involved in C, N, and S cycling^80^. The differential functional genes were ranked according to the Kruskal–Wallis test (*P* < 0.05).

A random forest model was used to predict the mean predictor importance for bacterial, fungal, and protistan communities, separately, as a driver for the functional genes in pathways of Xenobiotics Biodegradation and Metabolism. Then, key bacterial biomarkers involving the biodegradation process were identified according to the tenfold cross-validation error in using a random forest from the R package “*randomForest*”^81^. Phylogenetic relationships of a total of 56 bacterial biomarkers were constructed to form a phylogenetic tree (as a cladogram) by a maximum likelihood approach in the IQ-TREE method^82^. Phylogenetic trees were visualized using an online open-source tool (Interactive Tree of Life, version 4.4.2)^83^.

A Procrustes test in R software was conducted to evaluate the correlation between ARG profiles and microbial communities in deterministic and stochastic processes. Contribution (%) of deterministic and stochastic processes of bacterial, fungal, and protistan communities acting on ARGs profiles was assessed by VPA in the *vegan* and *labdsv* packages of R^68,84^. In order to clarify the influence of PE residues on the targeted parameters, the treatments without PE residues were combined for data analysis and represented by the abbreviation “–PE”, while treatments with PE residues (regardless of various PE concentrations) were combined and represented by the abbreviation “+PE” for comparison.

### Reporting summary

Further information on research design is available in the Nature Portfolio Reporting Summary linked to this article.

## Supplementary information

Supplementary information

Reporting Summary

## Data Availability

Raw sequencing data reported in this paper have been deposited in the Genome Sequence Read Archive in the National Genomics Data Center, China National Center for Bioinformation (GSA number, CRA015340). 16S rRNA amplicon sequencing data are available under Runs CRR1081112-CRR1081141 (Experiments CRX988007-CRX988036). ITS amplicon sequencing data are available under Runs CRR1081142-CRR1081171 (Experiments CRX988037-CRX988066). 18S rRNA amplicon sequencing data are available under Runs CRR1081220-CRR1081249 (Experiments CRX988115-CRX988144). Shotgun metagenomic sequencing data: Runs CRR1081097-CRR1081111 (Experiments CRX987992-CRX988006). Source data for graphs and charts presented in the study can be found at Figshare depository (10.6084/m9.figshare.29948720.v2)^85^.

## Code availability

The command line code of R software for the bioinformatics processing in this study was deposited in GitHub (https://github.com/chenyunshu0817/PE-residue2024) and Zenodo (10.5281/zenodo.16902894)^86^.
