# Supplementary material for: Long-term exposure to polyethylene restructures the multi-kingdom soil microbiota in maize fields
Source: Commun Biol. 2025 Nov 24;8:1722. doi: 10.1038/s42003-025-08899-8 (PMC12669691; doi:10.1038/s42003-025-08899-8)
Supplement: Supplementary file 1 — Supplementary information [file 42003_2025_8899_MOESM1_ESM.pdf]

Supplementary information

Long-term exposure to polyethylene restructures the multi-kingdom soil microbiota in maize fields

Zhen Shi[a,b], Li Xiong[a,b], Zhaojie Li[a,b], Farooq Shah[b,c], Xin Zhou[d], Qianhua Yuan[a,b], Bao–Luo Ma[e], Wei Wu[a,b,*]

[a]School of Breeding and Multiplication (Sanya Institute of Breeding and Multiplication), Hainan University, Sanya, China

[b]School of Tropical Agriculture and Forestry, Hainan University, Haikou, China

[c]Department of Agronomy, Abdul Wali Khan University Mardan, Khyber Pakhtunkhwa, Pakistan

[d]State Key Laboratory of Mycology, Institute of Microbiology, Chinese Academy of Sciences, Beijing, China

[e]Ottawa Research and Development Centre, Agriculture and Agri–Food Canada, 960 Carling Ave, Ottawa, Canada

*Corresponding author. Tel.: +86 0898 66273827

E–mail: weiwu@hainanu.edu.cn (W. Wu)

This supplementary file includes:

Figures S1 to S16

Table S1 to S5

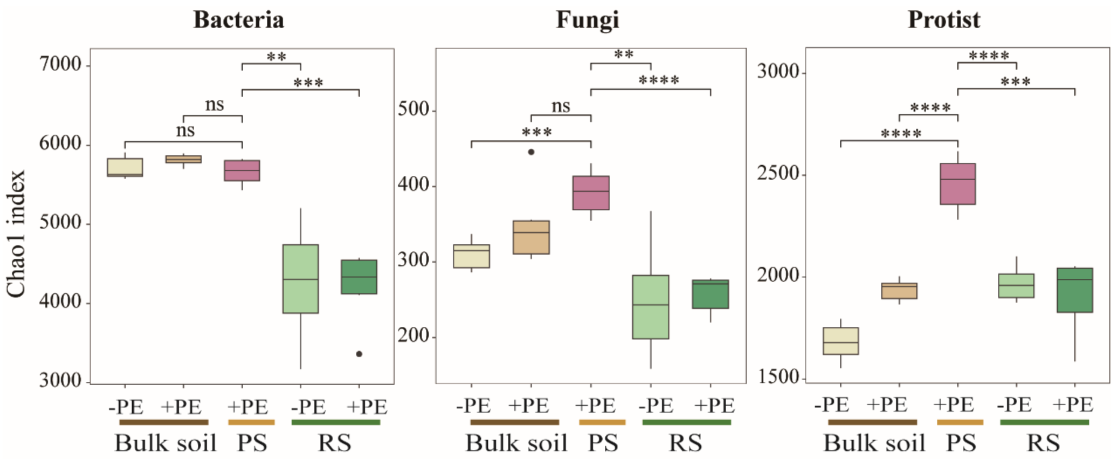

Figure S1. Alpha-diversity (Chao1 index) of bacterial, fungal, and protistan communities in response to PE residues (+PE) and without PE residues (-PE) in different compartment niches (BS: bulk soil; RS: rhizosphere soil; PS: plastisphere) (n=6). The top and bottom of the box represent the 75th and 25th quartiles, respectively, and the horizontal bars within them represent the average of each treatment. Black asterisks indicate significant difference ($^{*}p < 0.05$; $^{**}p < 0.01$; $^{***}p < 0.001$; $^{****}p < 0.0001$), while "ns" represents non-significant differences between different treatments using the Wilcoxon rank-sum test.

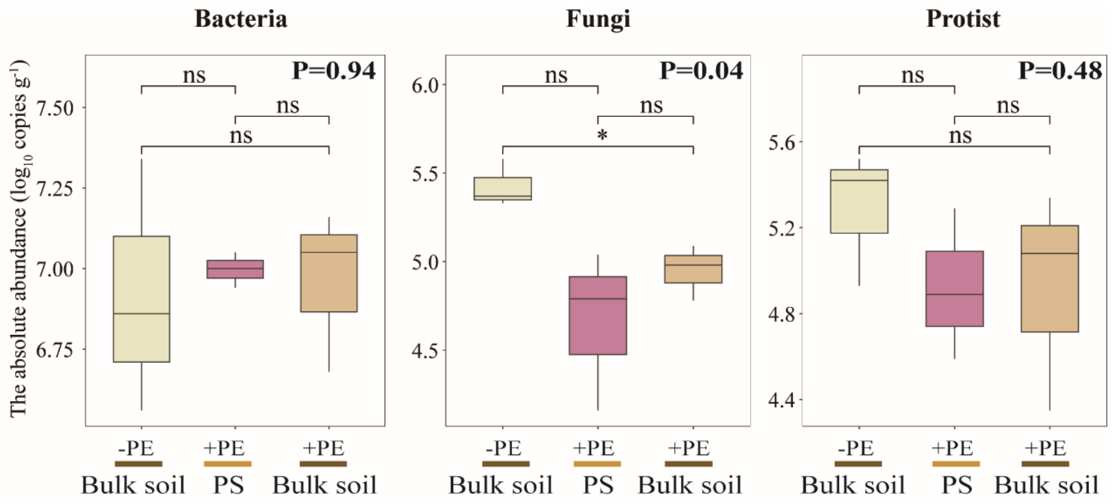

Figure S2. The absolute abundance (log_{10} gene copies per gram soil) of bacteria (16S rRNA gene), fungi (ITS region), and protists (18S rRNA gene) in response to PE residues (+PE) and without PE residues (-PE) in different compartment niches (BS: bulk soil; PS: plastisphere) (n=3). The top and bottom of the box represent the 75th and 25th quartiles, respectively, and the horizontal bars within them represent the average of each treatment. Black asterisks indicate significant difference ($^{*}p < 0.05$), while "ns" represents non-significant differences between different treatments using the Wilcoxon rank-sum test.

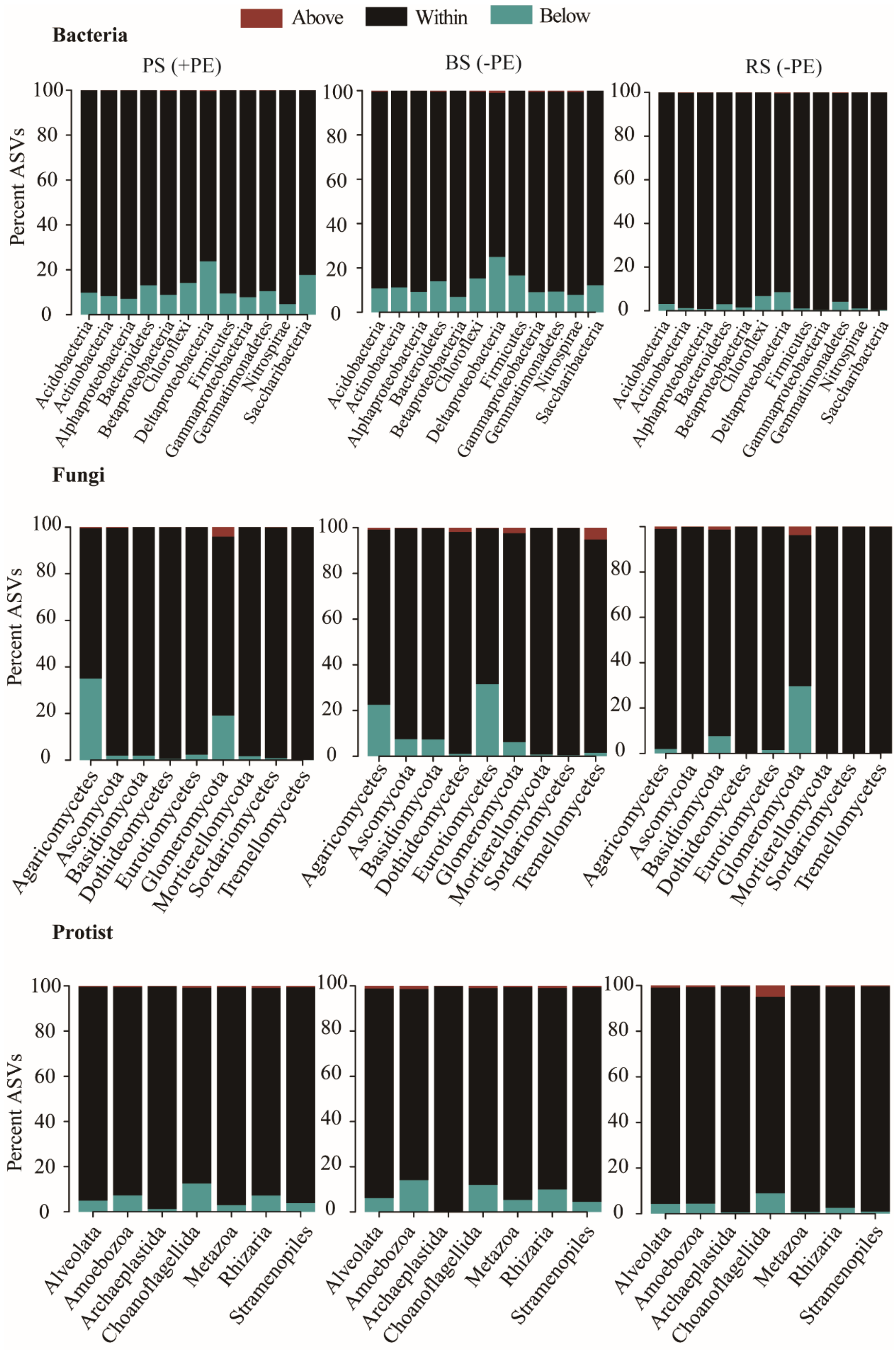

Figure S3. The percentage of ASVs at the phylum level that fall within, below, and above the 95% confidence interval of neutral model prediction for bacterial, fungal, and protistan communities in different compartment niches (BS: bulk soil; RS: rhizosphere soil; PS: plastisphere).

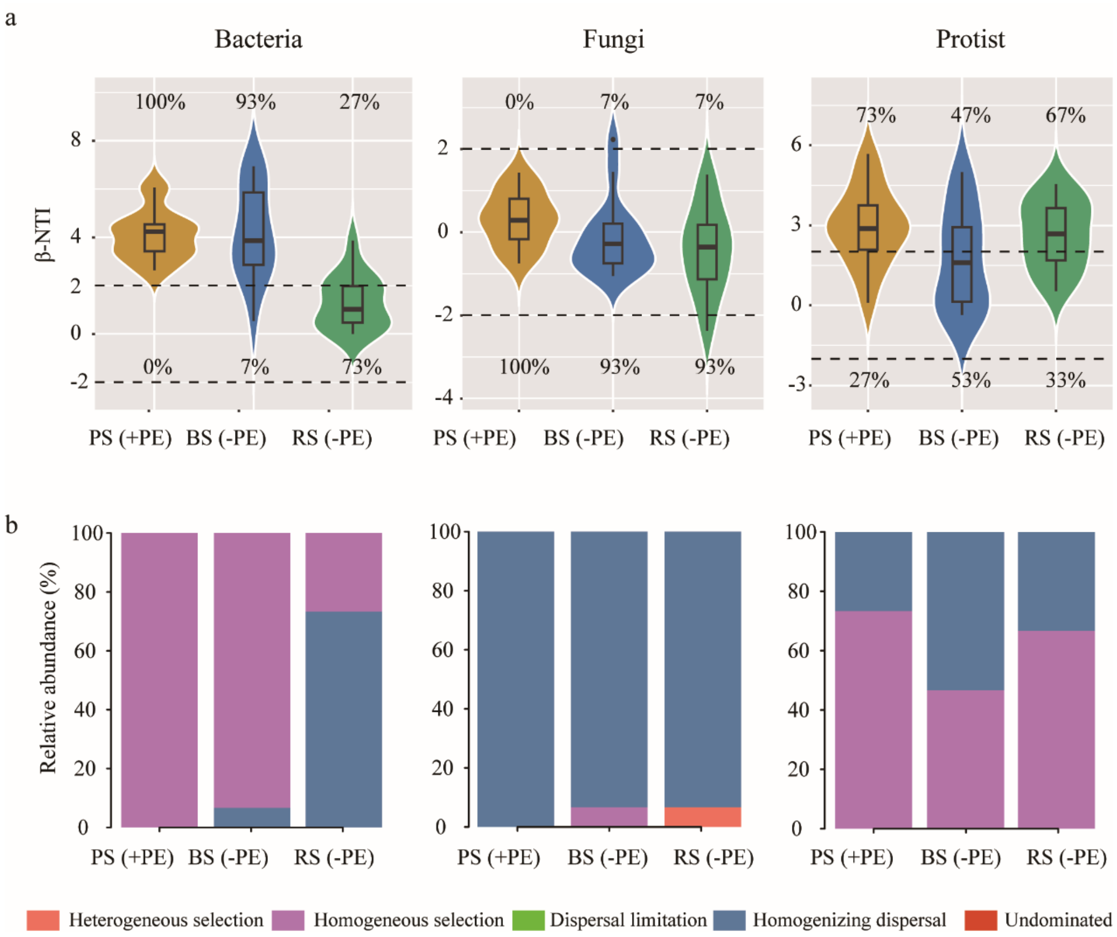

Figure S4. Deterministic and stochastic processes for bacterial, fungal and protistan communities in response to PE reside (+PE) and without PE reside (-PE) in different compartment niches (BS: bulk soil; RS: rhizosphere soil; PS: plastisphere). **(a)** Relative contributions of deterministic and stochastic processes to microbiome assembly, as inferred from β-Nearest Taxon Index (βNTI) values. βNTI was calculated using a null model with 999 randomizations. Values of $|\beta NTI| \geq 2$ indicate that deterministic processes dominate community assembly, whereas $|\beta NTI| < 2$ suggest stochastic processes are predominant. The percentages above and below each violin plot denote the proportion of deterministic and stochastic processes, respectively. **(b)** The relative importance of five ecological processes, i.e., heterogeneous selection, homogeneous selection, dispersal limitation, homogenizing dispersal, and undominated processes, based on βNTI and Bray–Curtis-based Raup-Crick Index (RCBray).

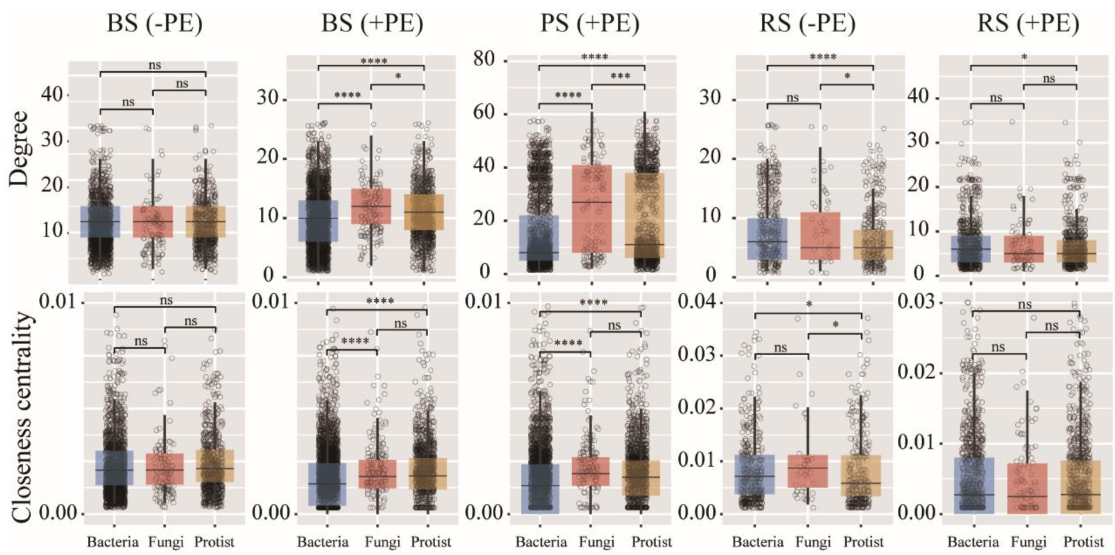

Figure S5. Comparison of node-level topological characteristics (degree and closeness centrality) between bacterial, fungal and protistan communities in response to PE reside (+PE) and without PE reside (-PE) under different compartment niches. Black asterisks indicate significant difference ($^{*}p < 0.05$; $^{**}p < 0.01$; $^{***}p < 0.001$; $^{****}p < 0.0001$), while "ns" represent non-significant differences between different treatments using the Wilcoxon rank-sum test.

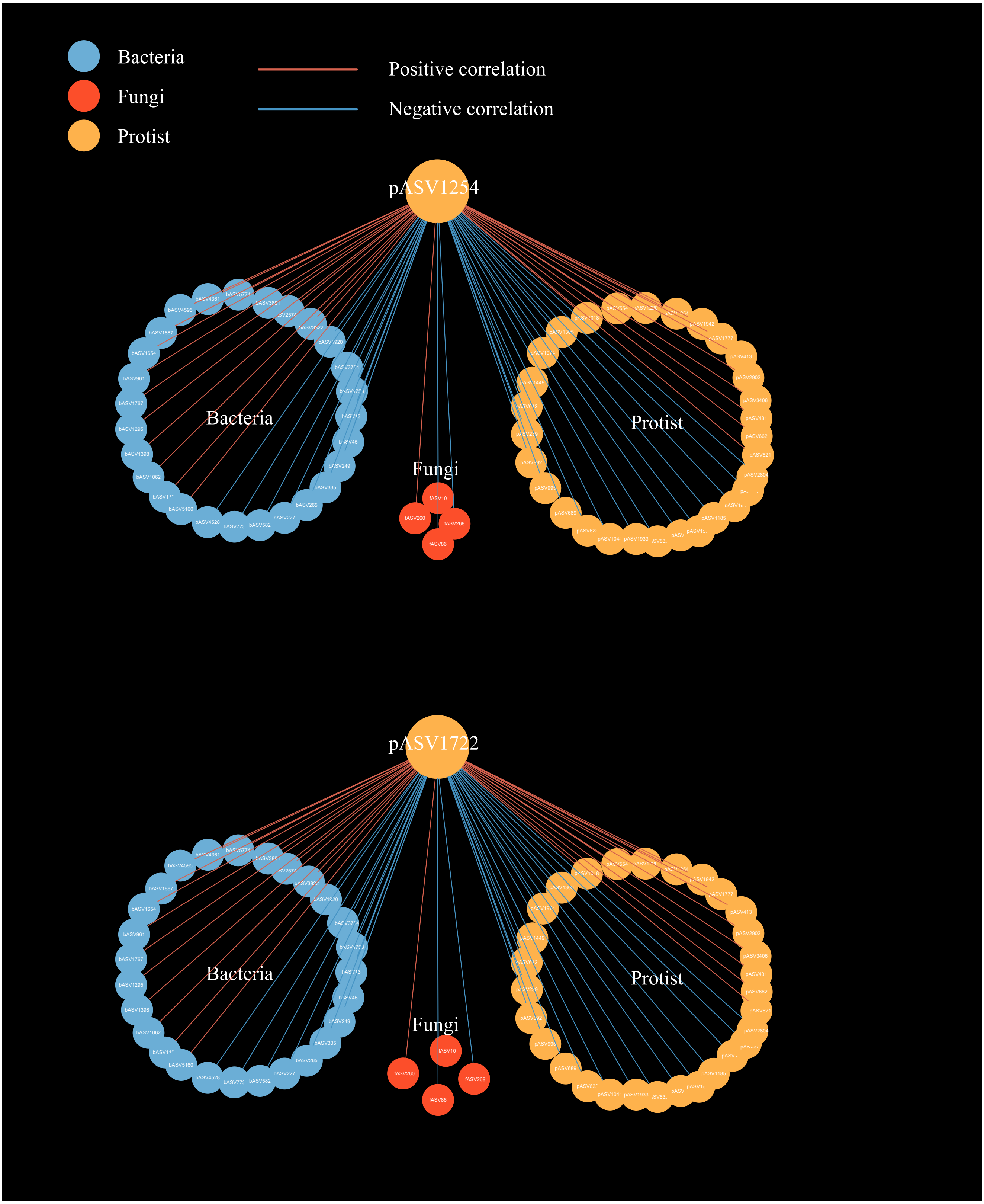

Figure S6. Co-occurrence network between two specific protistan ASVs of the genus Sandonidae and other bacterial and fungal taxa. Positive and negative collaborative relationships between different taxa are connected by red and blue lines, respectively. These two specific protistan ASVs were selected based on their highest node number, shown in the interkingdom co-occurrence network of Figure 3.

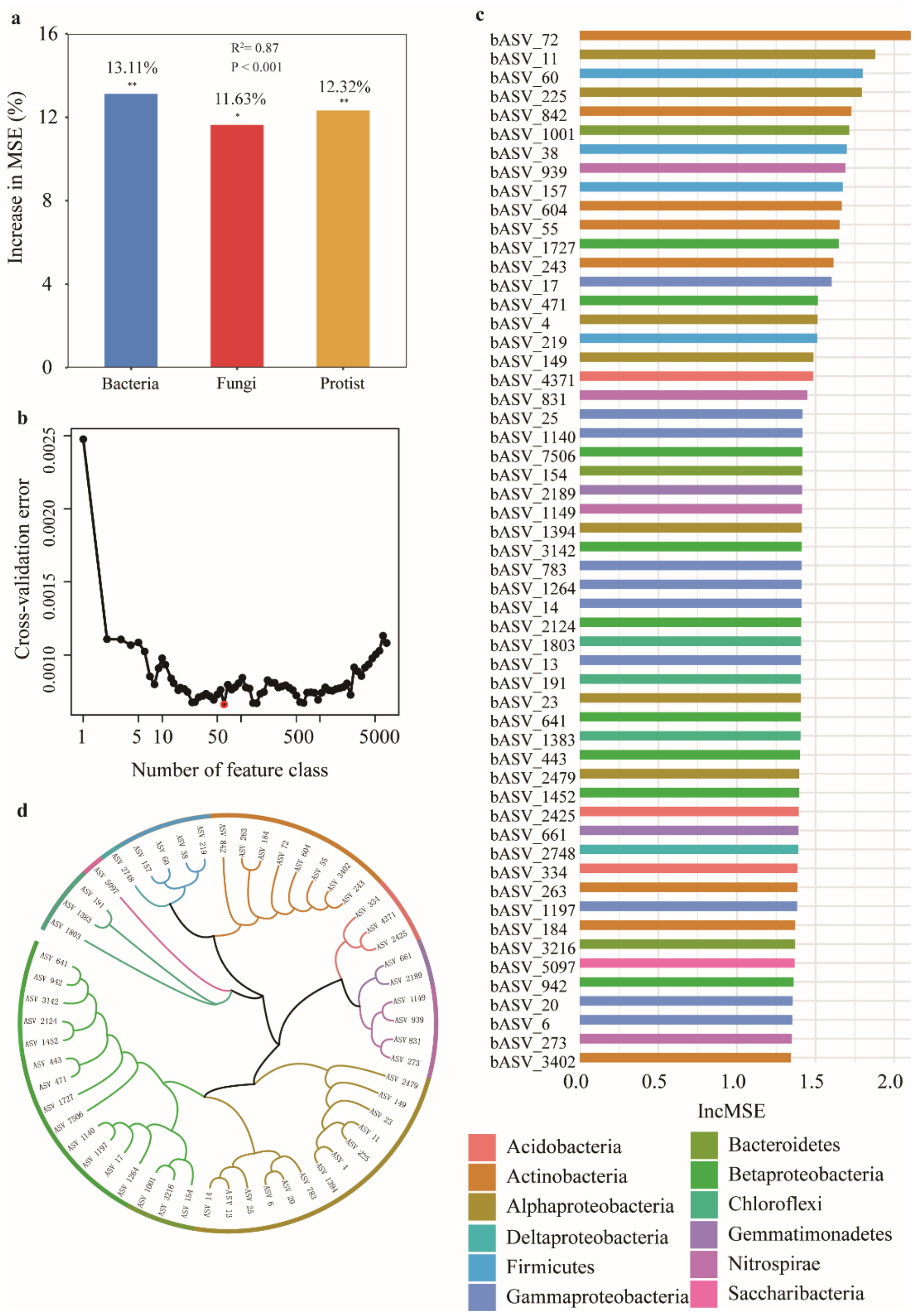

Figure S7. (a) Mean predictor importance (percentage of increase of mean square error) of bacterial, fungal, and protistan communities as drivers for the functional genes in the KEGG database involved with pathways of Xenobiotic Biodegradation and Metabolism, using a random forest. The accuracy predictor importance was computed for each tree and averaged over the forest (500 trees). Percentage increases in the MSE (mean squared error) of variables were used to estimate the importance of these predictors, and higher MSE% values imply more important predictors. **(b)** The tenfold cross-validation error and the identification of the key bacterial biomarkers that were involved in biodegradation. **(c)** Increase in the MSE of fifty-six bacterial biomarkers identified by random forest. **(d)** Cladogram illustrating phylogenetic relationships of 56 bacterial biomarkers involved with biodegradation.

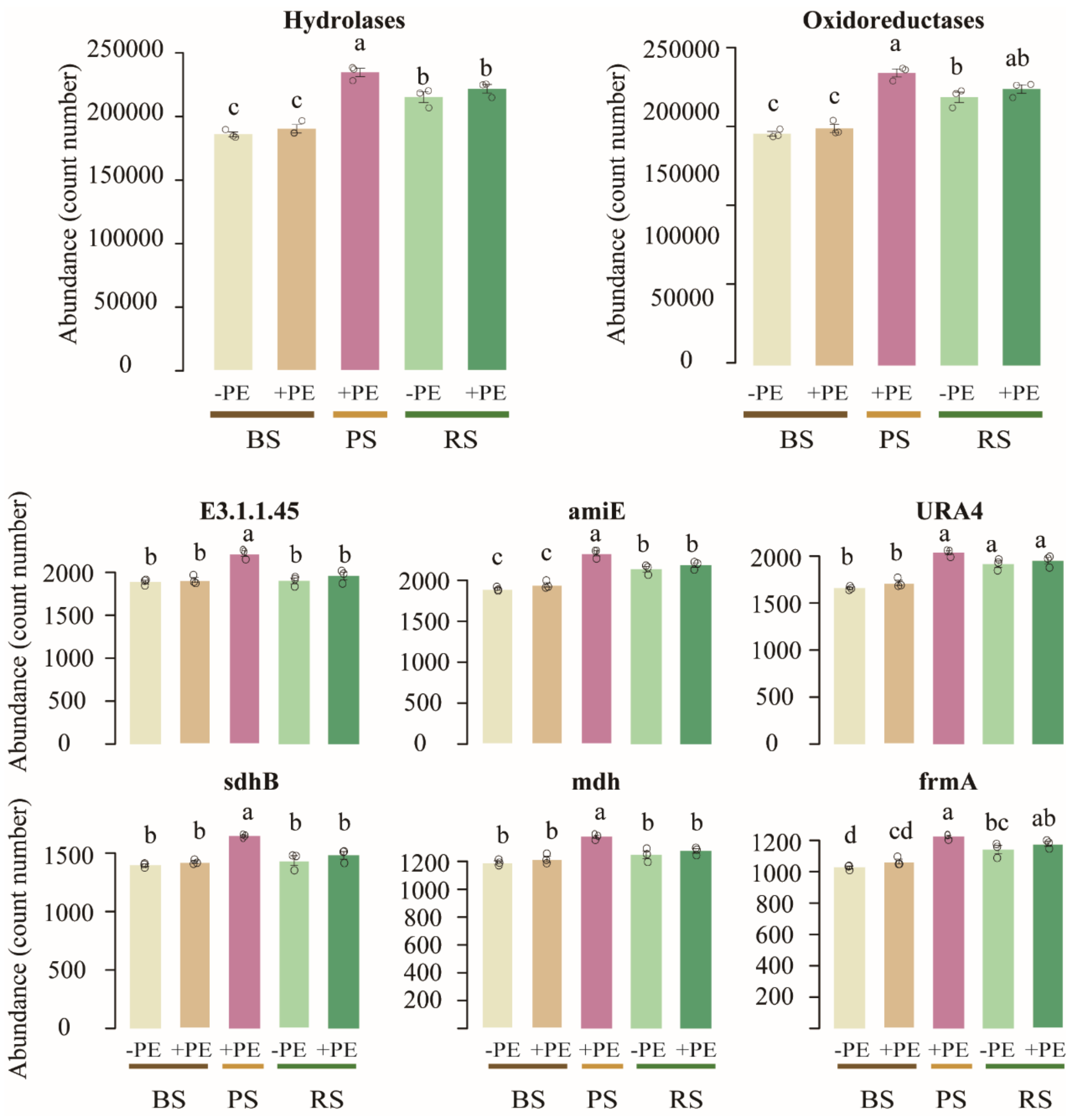

Figure S8. (a) The cumulative abundance (count number) of KEGG Orthology (KO) functional genes associated with the hydrolases and oxidoreductases in response to PE residues (+PE) and without PE residues (-PE) in different compartment niches (BS: bulk soil; PS: plastisphere; RS: rhizosphere soil). **(b)** The abundance of representative KO genes within the two enzyme classes. Selected KOs include specific hydrolases (e.g., E3.1.1.45, amiE, URA4) and oxidoreductases (e.g., sdhB, mdh, frmA), reflecting key microbial pathways involved in carbon degradation and redox metabolism. Treatments sharing the same letter are not significantly different, whereas those labeled with different letters differ significantly ($p < 0.05$). Error bars represent mean ± standard error (SE), with each data point indicating an individual sample library ($n = 3$).

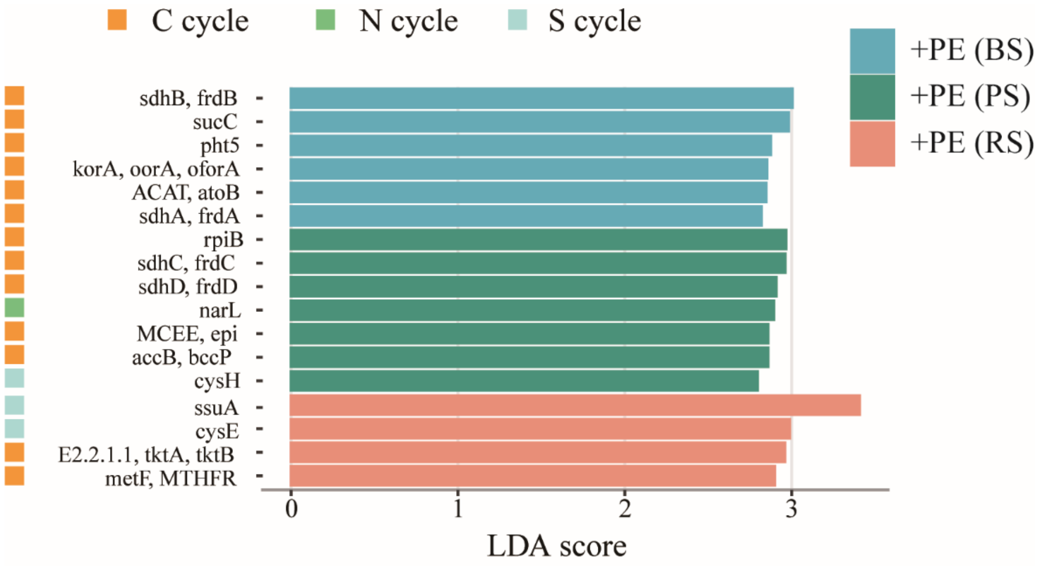

Figure S9. Identification of functional genes in the KO database involved with C, N, and S cycling using the linear discriminant analysis (LDA) effect size (LEfSe) in response to PE residue addition. Datasets were analyzed using the Kruskal-Wallis test ($P < 0.05$) to determine differential functional genes that were ranked according to the LDA score (log 10) (BS: bulk soil; PS: plastisphere; RS: rhizosphere soil).

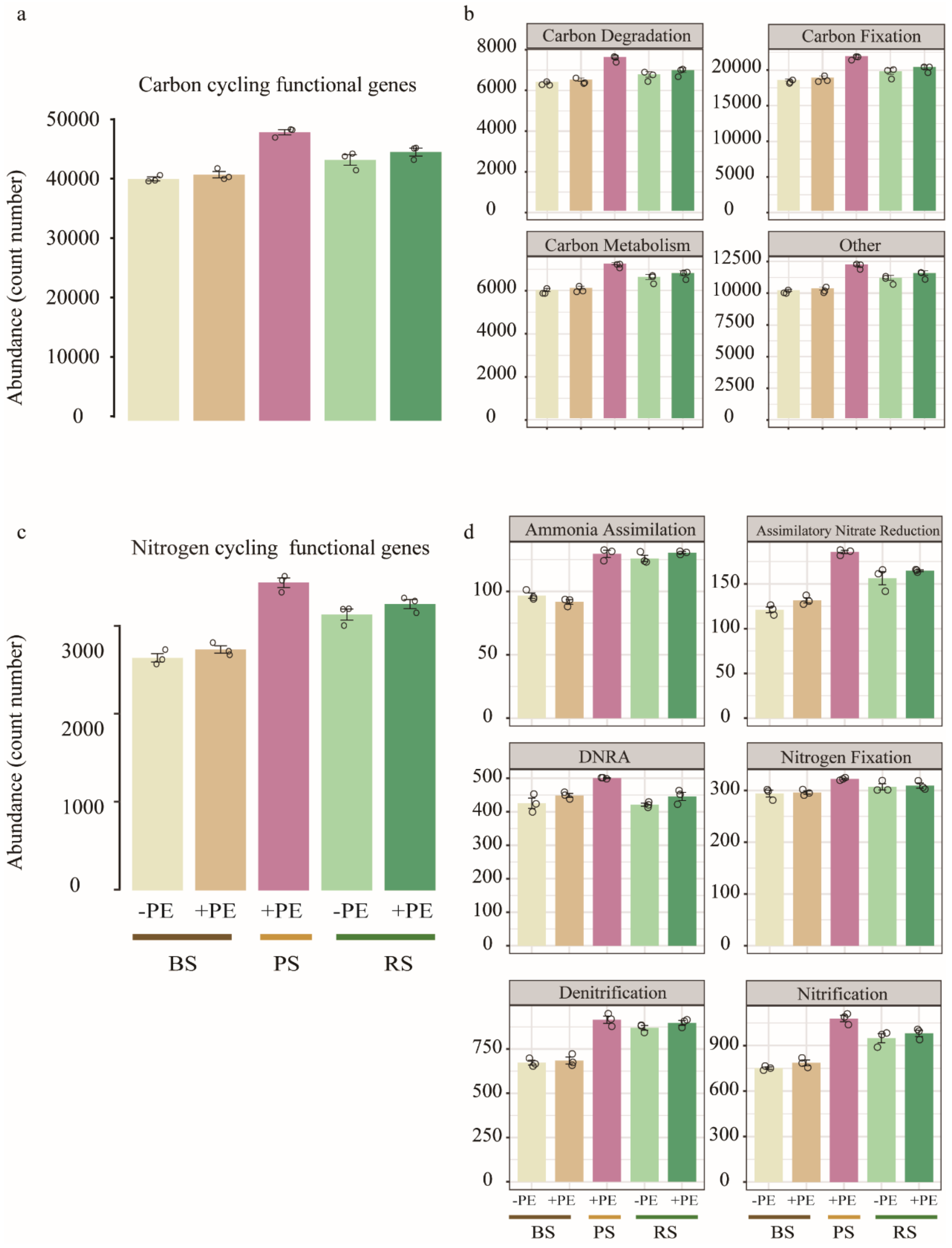

Figure S10. The abundance (count number) of KEGG Orthology (KO) functional genes associated with carbon cycling **(a)** and nitrogen cycling **(c)** under PE residues (+PE) and without PE residues (−PE) across different soil compartments (BS: bulk soil; PS: plastisphere; RS: rhizosphere). Panels **(b)** and **(d)** present the abundance of specific KO functional genes associated with carbon cycling **(b)** and nitrogen cycling **(d)** highlighs key microbial pathways impacted by PE exposure. DNRA: Dissimilatory Nitrate Reduction to Ammonium. Error bars represent mean ± standard error (SE), with each data point indicating an individual sample library (n = 3).

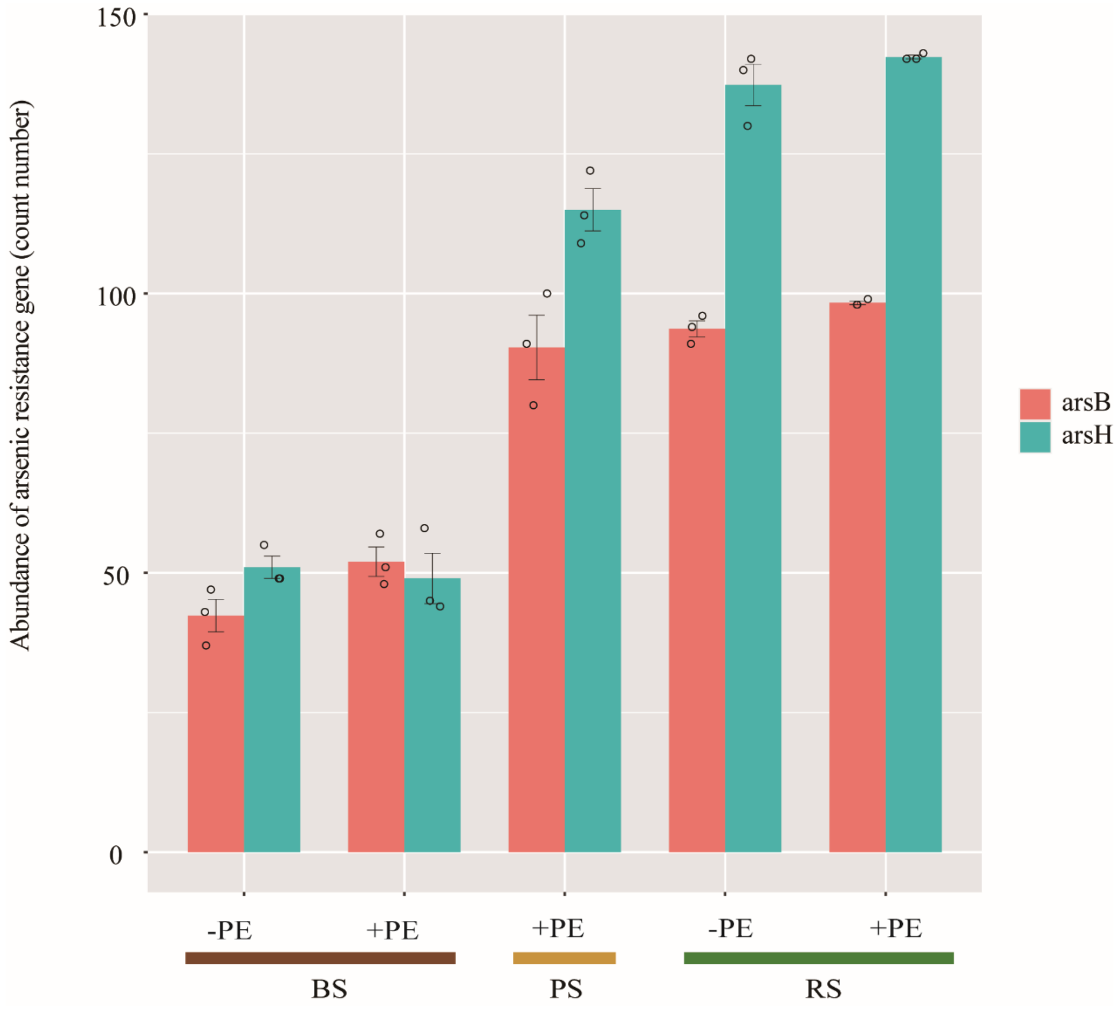

Figure S11. Abundance of arsenic resistance genes (arsB and arsH) in KEGG database in response to PE residues (+PE) and without PE residues (-PE) under different compartment niches (BS: bulk soil; PS: plastisphere; RS: rhizosphere soil). Error bars represent mean ± standard error (SE), with each data point indicating an individual sample library (n = 3).

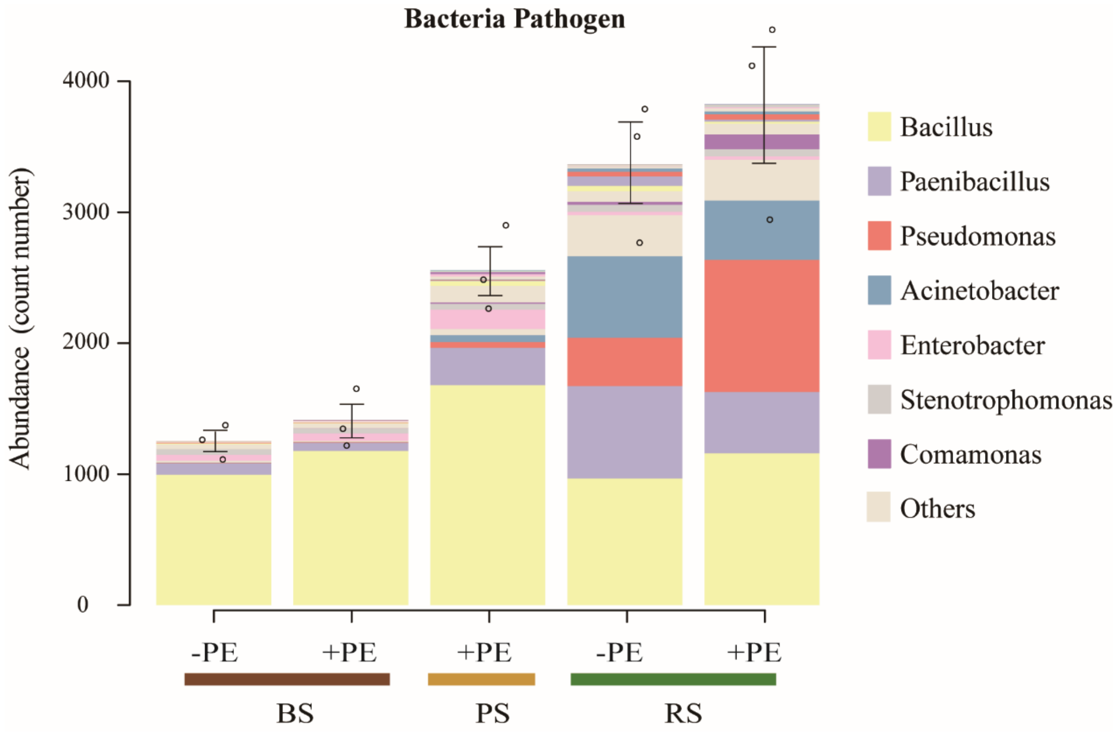

Figure S12. Potential pathogens (count number) in response to PE residues (+PE) and without PE residues (-PE) in different compartment niches (BS: bulk soil; PS: plastisphere; RS: rhizosphere soil). Error bars represent mean ± standard error (SE), with each data point indicating an individual sample library (n = 3).

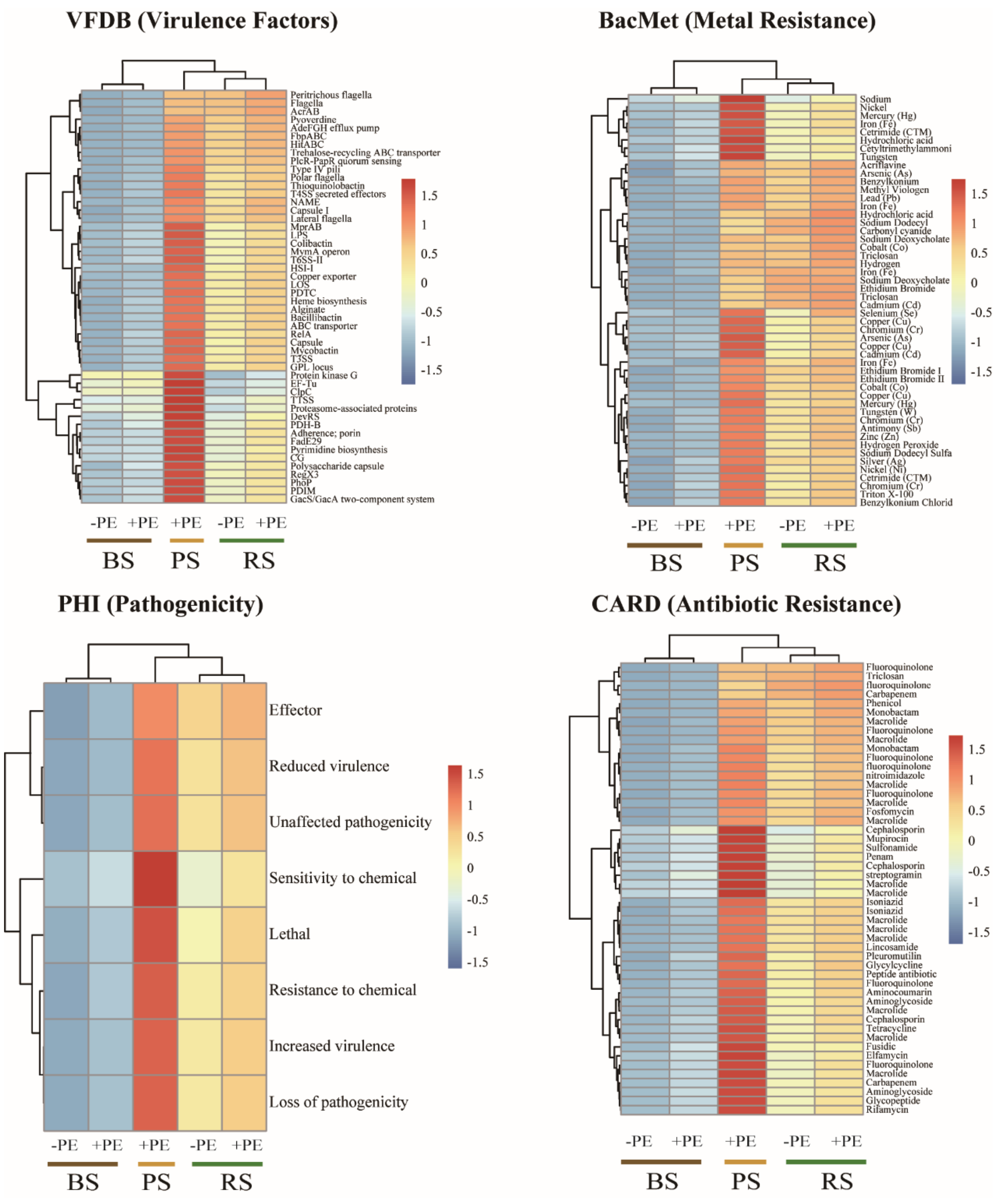

Figure S13. The top 50 most abundant functional genes (based on count number) annotated by the Virulence Factor Database (VFDB), Bacterial Metal Resistance Genes Database (BacMet), Pathogen–Host Interactions Database (PHI), and Comprehensive Antibiotic Resistance Database (CARD) under PE residues treatment (+PE) and without PE residues (−PE) across different soil compartments (BS: bulk soil; PS: plastisphere; RS: rhizosphere).

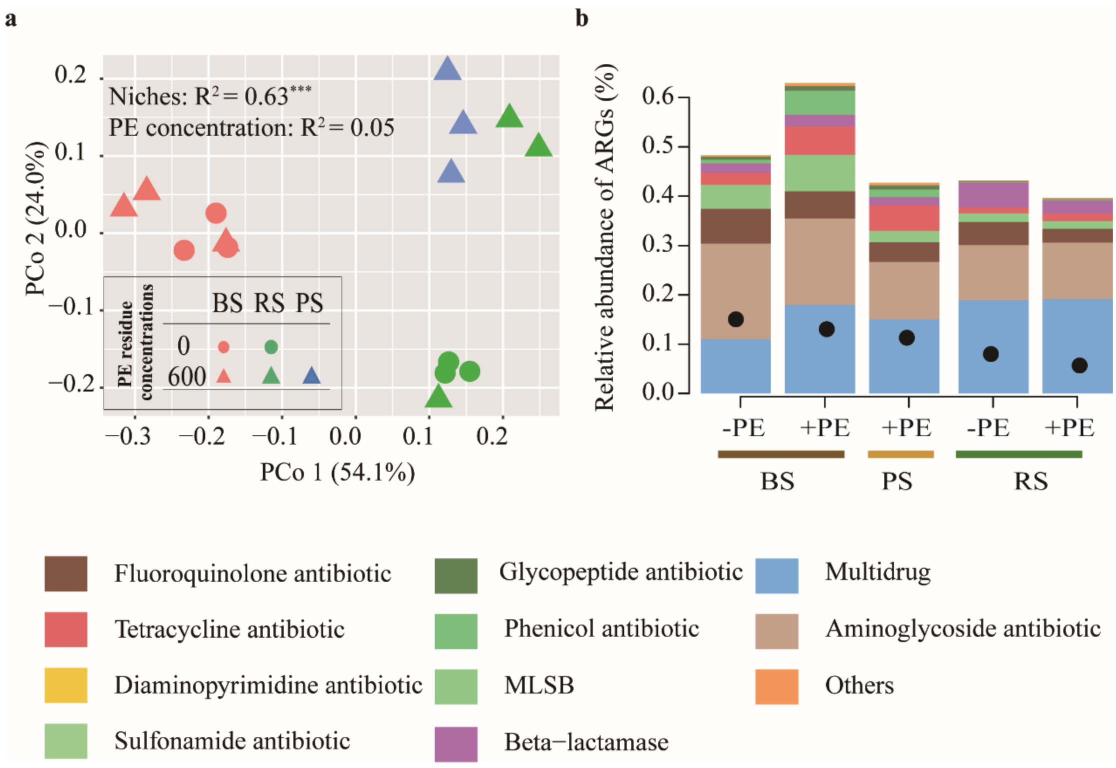

Figure S14. Relative abundance (%) of ARGs and mobile genetic elements (MGEs) **(a)**, and unconstrained PCoA ordinations based on the Bray-Curtis distance matrices **(b)** in response to PE residues (+PE) and without PE residues (-PE) in different compartment niches (BS: bulk soil; PS: plastisphere; RS: rhizosphere soil). A solid circle within a bar stands for MGEs. The percentage (%) on the X- and Y-axis of PCoA ordinations refers to the total variation explained.

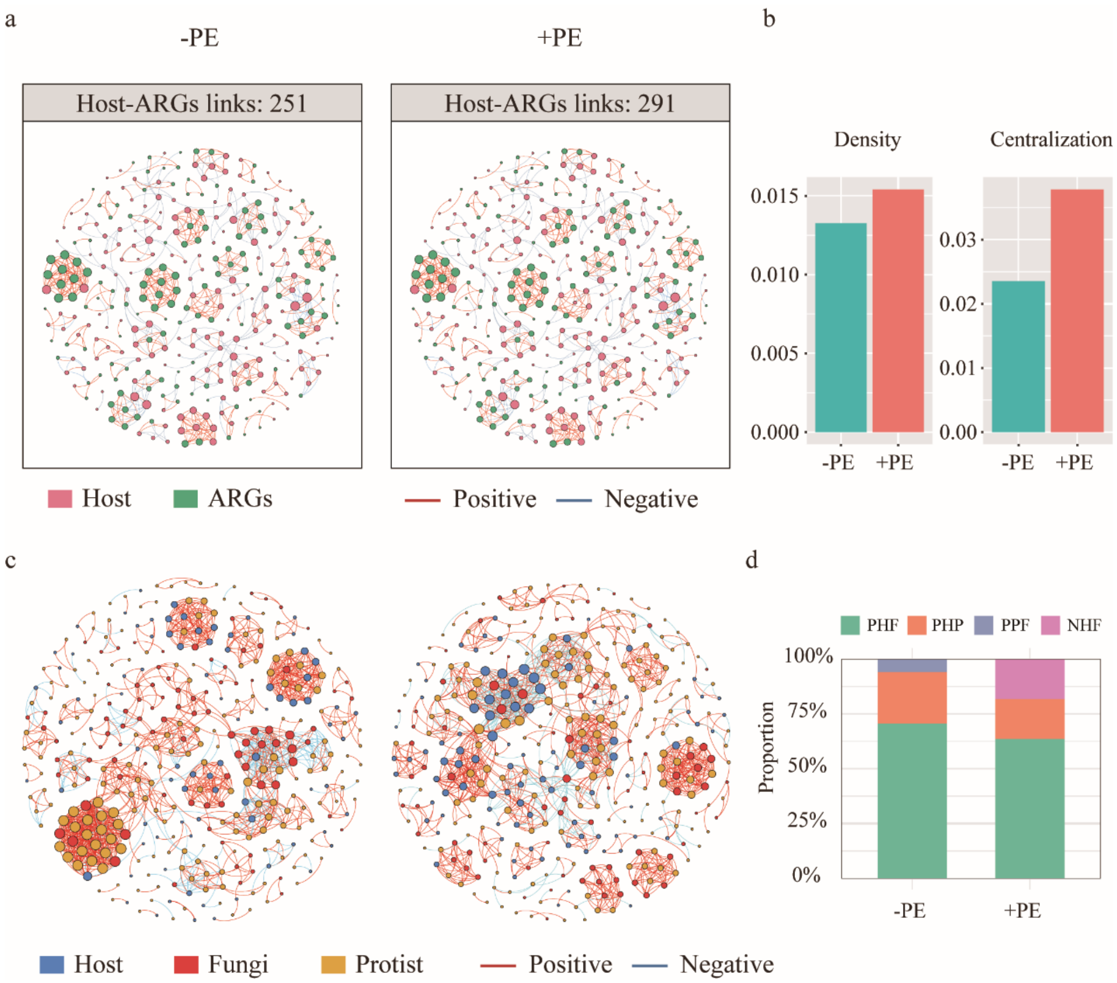

Figure S15. Co-occurrence of ARGs in association with multiple kingdoms in response to PE residues (+PE) and without PE residues (-PE). **(a)** Co-occurrence networks between ARGs (based on relative abundance) and bacterial community (based on the genus level, representing potential hosts). Edges represent significant correlations ($p < 0.001$), classified as positive (Spearman's $\rho > 0.7$; red) or negative (Spearman's $\rho < -0.7$; blue). **(b)** Comparison of topological characteristics of ARGs-host networks between the two treatments. **(c)** Co-occurrence networks between bacterial hosts, fungal, and protistan communities. Edges indicate significant correlations ($p < 0.001$), categorized as positive (red) or negative (blue) interactions based on Spearman's ρ values. **(d)** Proportions of positive and negative inter-kingdom associations between bacterial hosts, fungal and protistan genera within the network, including: PHF (positive host–fungi), PHP (positive host–protists), PPF (positive protist–fungi), and NHF (negative host–fungi) interactions.

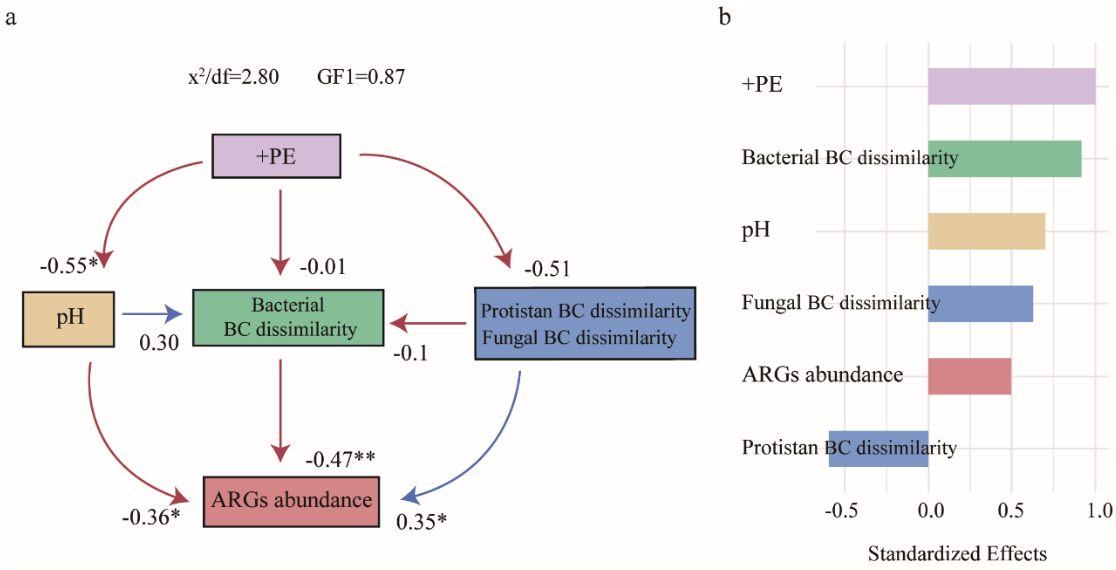

Figure S16. The influence of PE residues on soil pH, inter-kingdom microbial communities, and ARG abundance was evaluated using a structural equation model (SEM) (**a**), along with their corresponding standardized total effects (**b**). Microbial communities were quantified based on their Bray–Curtis dissimilarity. Arrows represent directional relationships from independent to dependent variables, as determined by the SEM models. Numbers adjacent to the arrows indicate the proportion of variance explained for each dependent variable. Asterisks denote significance levels (* $p < 0.05$; ** $p < 0.01$). Pathway colors indicate the positive (blue) and negative (red) relationships.

Table S1. Relative abundance (%) at phylum level for bacterial community as affected by different compartment niches and PE residue concentrations (0, 150, and 600 kg ha^{-1}).

	Gammaproteobacteria	Alphaproteobacteria	Actinobacteria	Acidobacteria	Betaproteobacteria	Chloroflexi	Bacteroidetes	Gemmatimonadetes	Deltaproteobacteria	Sacchribacteria	Firmicutes	Nitrosirae
Bulk soil												
CK	4.25a	8.76a	14.7a	27.4a	7.17a	10.3a	4.66a	7.36a	4.25a	1.04a	0.56a	2.94a
0	6.38a	10.9a	16.0a	21.9a	7.99a	10.3a	4.09a	7.17a	5.11a	0.83a	0.50a	3.55a
150	6.20a	10.6a	15.1a	22.5a	7.41a	11.0a	4.31a	7.14a	4.54a	1.25a	0.43a	3.17a
600	6.41a	11.3a	15.4a	21.5a	7.43a	10.7a	4.61a	7.83a	4.75a	1.48a	0.34a	3.14a
Mean	5.8	10.4	15.3	23.3	7.50	10.6	4.42	7.37	4.66	1.15	0.46	3.20
Rhizosphere soil												
CK	48.8a	18.6b	11.3a	2.35a	4.6b	0.88a	1.92a	0.54b	0.40c	3.78a	5.63a	0.22a
0	30.7a	25.2ab	12.2a	5.16a	10.3ab	1.86a	3.32a	1.12a	0.96a	4.04a	3.10b	0.56a
150	36.8a	28.9a	7.1a	4.78a	9.5ab	1.61a	3.63a	0.92ab	0.57bc	2.14a	2.86b	0.33a
600	29.7a	30.3a	10.3a	3.19a	11.3a	1.44a	4.73a	0.95ab	0.80ab	3.91a	1.96b	0.42a
Mean	36.5	25.8	10.2	3.87	8.9	1.45	3.40	0.88	0.68	3.47	3.39	0.38
Plasticsphere												
150	5.26b	15.9a	29.6a	16.5a	4.43a	10.1a	6.07a	4.32b	2.78a	1.25a	0.55b	1.01a
600	5.91a	13.3a	31.4a	15.8a	3.98b	10.7a	4.85b	4.65a	2.51a	1.24a	1.25a	1.40a
Mean	5.59	14.6	30.5	16.1	4.20	10.4	5.46	4.49	2.65	1.25	0.90	1.21

Table S2. Relative abundance (%) at phylum level for fungal community as affected by different compartment niches and PE residue concentrations (0, 150, and 600 kg ha^{-1}).

	Sordariomycetes	Mortierellomycota	Ascomycota	Glomeromycota	Agaricomycetes	Dothideomycetes	Eurotiomycetes	Basidiomycota	Tremellomycetes
Bulk soil									
CK	34.8a	24.5a	4.09a	21.8a	4.35b	2.38a	0.52a	5.90a	0.55a
0	24.1a	28.6a	5.47a	13.2a	19.8a	2.53a	1.17a	4.29a	0.24a
150	17.2a	22.4a	14.1a	24.1a	7.06ab	4.50a	3.81a	3.79a	0.31a
600	16.9a	36.0a	5.99a	26.5a	6.36ab	3.29a	0.95a	3.04a	0.07a
Mean	23.3	27.9	7.41	21.4	9.39	3.18	1.61	4.25	0.29
Rhizosphere soil									
CK	46.2a	33.7a	8.40b	0.08a	0.25b	8.25a	0.34a	0.15a	2.66a
0	31.0a	49.5a	9.24ab	0.31a	1.10b	4.61a	0.87a	0.20a	3.11a
150	49.6a	27.7a	9.72ab	0.22a	0.84b	5.44a	4.04a	0.11a	1.95a
600	41.9a	20.1a	17.1a	0.21a	9.16a	4.51a	3.19a	0.17a	2.85a
Mean	42.2	32.8	11.1	0.21	2.84	5.70	2.11	0.16	2.64
Plasticsphere									
150	31.9a	29.9a	11.4b	1.64a	5.79a	11.17a	1.57b	1.10a	3.74a
600	31.2a	28.7a	21.6a	1.65a	2.68b	5.88b	5.60a	0.50b	1.60a
Mean	31.6	29.3	16.5	1.65	4.23	8.52	3.58	0.80	2.67

Table S3. Relative abundance (%) at phylum level for protistan community as affected by different compartment niches and PE residue concentrations (0, 150, and 600 kg ha^{-1}).

	Metazoa	Rhizaria	Stramenopiles	Archaeplastida	Alveolata	Amoebozoa	Amoebozoa
Bulk soil							
CK	16.5a	26.5a	16.9a	24.0a	6.47a	6.04a	1.92a
0	7.51a	34.5a	19.3a	19.3a	5.85a	8.75a	2.68a
150	22.9a	27.6a	14.0a	18.7a	5.09a	7.93a	1.85a
600	24.1a	31.4a	14.2a	13.7a	5.74a	7.07a	1.73a
Mean	17.8	30.0	16.1	18.9	5.78	7.45	2.05
Rhizosphere soil							
CK	53.5a	10.9a	18.3a	6.11bc	5.33a	4.41a	1.01a
0	36.9a	13.3a	16.8a	22.1a	5.44a	4.10a	0.77a
150	46.5a	11.5a	22.7a	5.10c	5.98a	6.09a	1.23a
600	48.4a	10.4a	13.3a	16.6ab	4.59a	5.45a	0.75a
Mean	46.3	11.5	17.8	12.5	5.33	5.01	0.94
Plasticsphere							
150	20.6a	13.8a	18.0a	22.5a	15.0a	7.26a	1.37b
600	28.5a	15.8a	15.9a	12.4b	15.1a	7.69a	2.14a
Mean	24.5	14.8	17.0	17.4	15.1	7.48	1.75

Table S4. Soil environmental factors, including dissolved organic carbon (DOC, mg kg^{-1}); available nitrogen (AN, mg kg^{-1}); available phosphorus (AP, mg kg^{-1}); soil organic matter (SOM, g kg^{-1}), microbial biomass carbon (MBC, mg kg-1); microbial biomass nitrogen (MBN, mg kg^{-1}) under different PE residue concentrations.

PE residue concentration	DOC	AN	AP	SOM	MBC	MBN	pH
CK	92.3a	1.44b	7.27a	14.2a	730b	65.4b	8.14a
0	104a	1.99ab	7.24a	13.6a	976a	104a	7.91ab
150	79.6a	3.34ab	7.24a	15.2a	776ab	119a	7.79b
600	84.9a	4.04a	7.56a	15.7a	795ab	117a	7.88b

T**able S5**. The count number of functional genes involved with C, N, and S cycling in KEGG database in response to PE reside (+PE) and without PE reside (-PE) under different compartment niches.

**Gene names**	**Class**	**Bulk soil**		**PS**	**RS**	
		**-PE**	**+PE**	**+PE**	**-PE**	**+PE**
cysK	S	1344c	1398c	1635a	1530b	1576ab
nrnA	S	427bc	435b	483a	381d	407cd
metB	S	797c	823bc	974a	852bc	885b
sir	S	213b	217b	241a	209b	219ab
mddA	S	338b	340b	399a	332b	346b
cysNC	S	478b	495b	550a	494b	513ab
cysH	S	592c	611c	808a	730b	737b
ssuC	S	542b	552b	833a	867a	882a
soxC	S	255b	252b	280a	250b	252b
soxB	S	715c	738c	908a	835b	841b
soxA	S	138ab	136ab	150a	135ab	131b
ssuB	S	629b	630b	804a	823a	844a
tauD	S	907b	915b	1116a	1074a	1085a
cysD	S	861c	872c	1051a	969b	1018ab
soxD	S	161b	159b	184a	156b	156b
soxX	S	117b	119ab	142a	130ab	119ab
cysC	S	191b	193b	239a	209b	209b
soxY	S	175b	181b	235a	227a	222a
cysW	S	487b	495b	599a	573a	594a
soxA	S	98b	98b	119a	120a	121a
soxZ	S	114b	115b	136a	124ab	118ab
cysE	S	563b	576b	778a	755a	777a
metX	S	870c	899c	1100a	1015b	1052ab
cysL	S	43a	42a	52a	49a	46a
sbp	S	579c	582c	684a	648b	661ab
ssuA	S	926b	931b	1345a	1353a	1410a
cysU	S	470b	471b	564a	539a	560a
metZ	S	335b	341b	448a	438a	447a
dmdC	S	187b	188b	220a	213a	218a
cysA	S	491b	494b	569a	566a	572a
cysP	S	74c	77c	135b	156a	158a
soxB	S	64ab	65ab	67a	62b	64ab
SELENBP1	S	220ab	216b	232a	211b	215b
dmdB	S	186b	194b	234a	236a	242a
cysJ	S	179b	182b	305a	318a	327a
tauC	S	58b	60b	122a	135a	141a
soxD	S	76b	82b	127a	125a	129a
soeA	S	43.3bc	41.3c	50.7a	49ab	50.7a

dmsB	S	2.3b	2.3b	7a	9a	9.3a
sqr	S	199b	198b	254a	214b	219b
dmsC	S	1.33c	1c	4b	9a	9a
dmsC	S	2.67a	2.67a	3a	1.33a	1a
glnA	N	55a	54.7a	59a	57.7a	59.3a
nifH	N	2.33b	3.33ab	5ab	7.33a	7ab
nifD	N	2.33ab	2b	4ab	5a	4.67ab
nifK	N	2.33ab	2b	3.67ab	4.67ab	5a
nrfA	N	159ab	159ab	164a	145b	148b
nosZ	N	95b	95b	105a	94b	100ab
GDH2	N	128b	130b	146a	145a	145a
arcC	N	275c	292bc	357a	300bc	318b
narL	N	527c	549c	754a	625b	648b
nirK	N	275b	299ab	337a	270b	287b
cynS	N	71.3b	68b	89.7a	92a	94.7a
nasA	N	221b	230b	285a	283a	285a
nirD	N	294b	290b	433a	391a	413a
napB	N	41b	43.3b	62a	63.7a	65.7a
nirS	N	8.7b	9.3ab	11.7ab	12a	12a
norB	N	108.3b	118.3b	140a	136.3a	137a
cah	N	117b	119b	185a	188a	196a
cah	N	86c	98c	137a	115b	126ab
nirA	N	56b	59ab	62a	57b	59ab
napA	N	51b	53b	62a	63a	63a
norC	N	25b	24b	40a	34a	36a
nasB	N	35c	34c	49a	41b	39bc
fadN	C	445b	451b	520a	504a	515a
cysK	C	1344c	1398c	1635a	1530b	1576ab
folD	C	1073b	1075b	1287a	1127b	1169b
accC	C	1144d	1151cd	1263a	1199bc	1216ab
sucC	C	1395c	1440bc	1629a	1461bc	1516b
glk	C	843b	899b	1077a	833b	881b
glk	C	544b	567b	801a	738a	779a
aceE	C	332b	332b	366a	358a	358a
mdh	C	1192b	1218b	1378a	1247b	1275b
ppc	C	203b	210b	251a	251a	250a
ppdK	C	208b	208b	222a	214ab	214b
dld	C	12b	14b	25a	28a	28a
dld	C	9b	13b	24a	28a	28a
gapdh	C	19b	16b	25a	28a	28a
