# Supplementary material for: Long-term exposure to polyethylene restructures the multi-kingdom soil microbiota in maize fields
Source: Commun Biol. 2025 Nov 24;8:1722. doi: 10.1038/s42003-025-08899-8 (PMC12669691; doi:10.1038/s42003-025-08899-8)
Supplement: Supplementary file 2 — Reporting Summary [file 42003_2025_8899_MOESM2_ESM.pdf]

nature portfolio

Corresponding author(s): Wei Wu

Last updated by author(s): Aug 23, 2025

Reporting Summary

Nature Portfolio wishes to improve the reproducibility of the work that we publish. This form provides structure for consistency and transparency in reporting. For further information on Nature Portfolio policies, see our Editorial Policies and the Editorial Policy Checklist.

Statistics

For all statistical analyses, confirm that the following items are present in the figure legend, table legend, main text, or Methods section.

n/a	Confirmed	
☐	☒	The exact sample size (*n*) for each experimental group/condition, given as a discrete number and unit of measurement
☐	☒	A statement on whether measurements were taken from distinct samples or whether the same sample was measured repeatedly
☐	☒	The statistical test(s) used AND whether they are one- or two-sided<br>*Only common tests should be described solely by name; describe more complex techniques in the Methods section.*
☒	☐	A description of all covariates tested
☐	☒	A description of any assumptions or corrections, such as tests of normality and adjustment for multiple comparisons
☐	☒	A full description of the statistical parameters including central tendency (e.g. means) or other basic estimates (e.g. regression coefficient) AND variation (e.g. standard deviation) or associated estimates of uncertainty (e.g. confidence intervals)
☒	☐	For null hypothesis testing, the test statistic (e.g. *F*, *t*, *r*) with confidence intervals, effect sizes, degrees of freedom and *P* value noted<br>*Give P values as exact values whenever suitable.*
☒	☐	For Bayesian analysis, information on the choice of priors and Markov chain Monte Carlo settings
☒	☐	For hierarchical and complex designs, identification of the appropriate level for tests and full reporting of outcomes
☐	☒	Estimates of effect sizes (e.g. Cohen's *d*, Pearson's *r*), indicating how they were calculated

Our web collection on statistics for biologists contains articles on many of the points above.

Software and code

Policy information about availability of computer code

Data collection	No special software was used to collect data.
Data analysis	The R scriptis is available in a publicly accessible database (https://github.com/chenyunshu0817/PE-residue2024).<br>fastp(v.0.20.0), FLASH, USEARCH(v.10.0), VSEARCH(v.2.1.4), DADA2, SILVA(v13.8), UNITE(v8.0) and PR2 (v4.12.0) were used for amplicon analyses.<br>MEGAHIT(v.1.0.6), MetaGeneMark(v.3.38), CD-HIT(v.4.7), Bowtie2(v.2.4.2), DIAMOND(v.2.0.14) and BLASTX were used for metagenome analyses.<br>R software(v.4.2.0), Microsoft Excel 2016 and Adobe Illustrator 2020 were used for data analysis and visualization in the manuscript.

For manuscripts utilizing custom algorithms or software that are central to the research but not yet described in published literature, software must be made available to editors and reviewers. We strongly encourage code deposition in a community repository (e.g. GitHub). See the Nature Portfolio guidelines for submitting code & software for further information.

Data

Policy information about availability of data

All manuscripts must include a data availability statement. This statement should provide the following information, where applicable:

- Accession codes, unique identifiers, or web links for publicly available datasets
- A description of any restrictions on data availability
- For clinical datasets or third party data, please ensure that the statement adheres to our policy

The sequencing data generated in this study have been deposited in the National Genomics Data Center, China National Center for Bioinformation (GSA number, 15340). Source data are provided with this paper.

Research involving human participants, their data, or biological material

Policy information about studies with human participants or human data. See also policy information about sex, gender (identity/presentation), and sexual orientation and race, ethnicity and racism.

Reporting on sex and gender	not applicable
Reporting on race, ethnicity, or other socially relevant groupings	not applicable
Population characteristics	not applicable
Recruitment	not applicable
Ethics oversight	not applicable

Note that full information on the approval of the study protocol must also be provided in the manuscript.

Field-specific reporting

Please select the one below that is the best fit for your research. If you are not sure, read the appropriate sections before making your selection.

☐ Life sciences ☐ Behavioural & social sciences ☒ Ecological, evolutionary & environmental sciences

For a reference copy of the document with all sections, see nature.com/documents/nr-reporting-summary-flat.pdf

Ecological, evolutionary & environmental sciences study design

All studies must disclose on these points even when the disclosure is negative.

Study description	This study explores the effects of polyethylene (PE) residues on the interactions between multi-kingdom domains (bacteria, fungi, and protists), and the antibiotic resistome. The experimental treatments were initiated in 2014 and maintained in each plot for long–term observation until 2021, with three distinct PE residue concentrations (0, 150, and 600 kg PE ha^{-1}). In 2021, samples were collected from three soil compartment niches (bulk soil, rhizosphere, and plastisphere) at the maturity stage. The specific objectives of this study were to: (1) characterize the diversity, composition, assembly, and functional profiles of multi-kingdom soil microbiota following long–term exposure to PE residues; (2) investigate inter-kingdom interactions through integrated co-occurrence network analysis; and (3) identify the composition of the antibiotic resistome, and the potential microbial pathogens, and examine their relationships with the multi-kingdom microbiota.
Research sample	There are 30 samples for amplicon analysis of bacteria, fungi, and protists, considering varying levels of PE residue concentrations (including 0, 150, and 600 kg PE ha^{-1}) and different niche compartments (bulk soil, rhizosphere, and plastisphere). For the analysis of the composition of antibiotic resistome following long-term PE residue exposure, we selected 15 samples, considering two levels of PE residue and two niche compartments. These samples were analyzed using high-throughput qPCR techniques and metagenomic sequencing.
Sampling strategy	As of the 2021 sampling year, PE residues had been embedded in the topsoil for eight consecutive years, allowing us to assess their legacy effects. Soil samples were collected in 2021 from three distinct compartment niches (bulk soil, rhizosphere, and plastisphere) at the maturity stage. Bulk soil samples were obtained from the topsoil between maize plant rows using a stainless-steel auger. Rhizosphere soil, which was tightly adhered to fine roots, was carefully sampled from each plot. For plastisphere samples, plastic residues were manually retrieved from the topsoil, and the soil tightly bound to the film surface was extracted by immersing the plastic in a sterile saline solution. The resulting soil suspension was then centrifuged at 10,000 ×g for 10 minutes, and the pellet obtained was defined as plastisphere soil.
Data collection	Soil samples were collected and stored at –80 ℃ before DNA extraction. Soil genomic DNA was extracted from 0.5 g of fresh soil

	using the FastDNA™ SPIN Kit. Soil physicochemical properties were measured and recorded by Zhen Shi. Amplicon and metagenomic sequencing were conducted at Magigen Co., Ltd. (Guangzhou, China) using Illumina sequencing platforms.
Timing and spatial scale	Field experiments were conducted from 2014 to 2021 at the Changwu Agro-Ecological Research Station, located in the Loess Plateau of Shaanxi Province, northwest China (107°40′E, 35°12′N). Maize was planted each year in April and harvested in September, after which the field was left fallow until the following April. In the 2021 sampling year, PE residues had been embedded in the topsoil for eight consecutive years. Soil samples were collected at the maize maturity stage in the final year to evaluate their long-term legacy effects on the soil microbiome.
Data exclusions	No data were excluded from the analyses.
Reproducibility	The results can be reproduced
Randomization	The field experiments were performed according to a randomized complete block design
Blinding	All sample processing was conducted without any signs or labels indicating the corresponding treatments to ensure unbiased handling

Did the study involve field work? ☒ Yes ☐ No

Field work, collection and transport

Field conditions	The experimental site was characterized by a dry semi-humid climate. The annual average precipitation and air temperature from 2020 to 2021 were 584.1 mm and 10.1 oC, respectively. The experimental field had dark loessal soil. In the 2021 sampling year, the topsoil (0–20 cm) contained 14.7 g kg–1 soil organic matter, 2.70 mg kg–1 available N, 7.33 mg kg–1 available P, with a pH of 7.93.
Location	Field experiments were executed from 2014 to 2021 at the Changwu Agro-Ecological Research Station, located in the Loess Plateau of Shaanxi province, northwest China (107°40 E, 35°12 N).
Access & import/export	The samples were placed in sterile plastic bags and rapidly transported on ice to the laboratory at Hainan University.
Disturbance	This study did not cause any environmental disturbance.

Reporting for specific materials, systems and methods

We require information from authors about some types of materials, experimental systems and methods used in many studies. Here, indicate whether each material, system or method listed is relevant to your study. If you are not sure if a list item applies to your research, read the appropriate section before selecting a response.

Materials & experimental systems

n/a	Involved in the study	
☒	☐	Antibodies
☒	☐	Eukaryotic cell lines
☒	☐	Palaeontology and archaeology
☒	☐	Animals and other organisms
☒	☐	Clinical data
☒	☐	Dual use research of concern
☐	☒	Plants

Methods

n/a	Involved in the study	
☒	☐	ChIP-seq
☒	☐	Flow cytometry
☒	☐	MRI-based neuroimaging

Dual use research of concern

Policy information about dual use research of concern

Hazards

Could the accidental, deliberate or reckless misuse of agents or technologies generated in the work, or the application of information presented in the manuscript, pose a threat to:

No | Yes

- [x] No [ ] Yes — Public health
- [x] No [ ] Yes — National security
- [x] No [ ] Yes — Crops and/or livestock
- [x] No [ ] Yes — Ecosystems
- [x] No [ ] Yes — Any other significant area

Experiments of concern

Does the work involve any of these experiments of concern:

No | Yes

- [x] No [ ] Yes — Demonstrate how to render a vaccine ineffective
- [x] No [ ] Yes — Confer resistance to therapeutically useful antibiotics or antiviral agents
- [x] No [ ] Yes — Enhance the virulence of a pathogen or render a nonpathogen virulent
- [x] No [ ] Yes — Increase transmissibility of a pathogen
- [x] No [ ] Yes — Alter the host range of a pathogen
- [x] No [ ] Yes — Enable evasion of diagnostic/detection modalities
- [x] No [ ] Yes — Enable the weaponization of a biological agent or toxin
- [x] No [ ] Yes — Any other potentially harmful combination of experiments and agents

Plants

Seed stocks	This study does not include seed stock
Novel plant genotypes	This study does not involve any novel plant genotypes
Authentication	n/a
